# Fatigue during treatment for hepatitis C virus: results of self-reported fatigue severity in two Phase IIb studies of simeprevir treatment in patients with hepatitis C virus genotype 1 infection

**DOI:** 10.1186/1471-2334-14-465

**Published:** 2014-08-26

**Authors:** Jane Scott, Kathleen Rosa, Min Fu, Karin Cerri, Monika Peeters, Maria Beumont, Stefan Zeuzem, Donna M Evon, Leen Gilles

**Affiliations:** Janssen Global Services LLC, 50-100 Holmers Farm Way, Holmers Lane, High Wycombe, Bucks, HP12 4DP UK; University of North Carolina at Wilmington, Wilmington, NC USA; Janssen Research and Development, Pennsylvania, PA USA; Janssen Pharmaceutical NV, Beerse, Belgium; Janssen Research and Development, Beerse, Belgium; Klinikum der Johann-Wolfgang-Goethe-Universität–Med. Klinik I, Frankfurt, Germany; Division of Gastroenterology and Hepatology, University of North Carolina, Chapel Hill, NC USA

**Keywords:** EQ-5D, Fatigue, FSS, Hepatitis C virus, Patient-reported outcomes, Peginterferon-α, Ribavirin, Simeprevir, TMC435, Triple therapy

## Abstract

**Background:**

Fatigue is a common symptom of chronic hepatitis C virus (HCV) infection and a frequent side-effect of peginterferon/ribavirin (PR) therapy for HCV. This study evaluated the impact of adding the oral HCV NS3/4A protease inhibitor simeprevir to PR on patient-reported fatigue and health status among patients with chronic HCV genotype 1 infection enrolled in the Phase IIb PILLAR and ASPIRE trials [NCT00882908; NCT00980330].

**Methods:**

Treatment-naïve patients (PILLAR, n = 386) and treatment-experienced patients (ASPIRE, n = 462) were randomized to simeprevir plus PR (simeprevir/PR) or placebo plus PR (placebo/PR). In PILLAR, duration of PR treatment in the simeprevir/PR groups was determined using response-guided therapy (RGT) criteria. PR could be terminated at Week 24, instead of Week 48, if HCV RNA was <25 IU/mL by Week 4 and then undetectable at Weeks 12, 16, and 20. In both studies, patients completed the Fatigue Severity Scale (FSS) and EQ-5D quality-of-life questionnaire in their native language at baseline and throughout the studies up until Week 72.

**Results:**

During the first 24 weeks of treatment, mean FSS total score was increased to a similar degree compared with baseline among patients receiving simeprevir/PR or placebo/PR in both studies indicating increased fatigue severity. Mean FSS scores returned to values comparable with baseline among patients receiving simeprevir/PR after Week 24 in PILLAR (after treatment completion for the majority of patients) and in ASPIRE (after Week 48), consistent with RGT enabling early termination of all treatment at Week 24 in 82.2% of simeprevir/PR-treated patients in the PILLAR study. Similar results were observed for EQ-5D, with simeprevir/PR-treated patients experiencing less time with worse health problems according to EQ-5D scores compared with placebo/PR groups in both studies, and more rapid improvement in health status associated with shorter treatment duration in the PILLAR study.

**Conclusions:**

Combination of simeprevir with PR did not increase patient-reported fatigue severity or health status impairments beyond that reported by patients treated with PR alone. Many patients treated with simeprevir/PR returned to pretreatment fatigue and health status levels sooner due to increased treatment efficacy that enabled shorter duration of all therapy, compared with PR alone.

**Electronic supplementary material:**

The online version of this article (doi:10.1186/1471-2334-14-465) contains supplementary material, which is available to authorized users.

## Background

Hepatitis C virus (HCV) is a major public health challenge. Recent estimates by the World Health Organization suggest that approximately 130–170 million people are chronically infected with HCV worldwide and more than 350000 people are estimated to die from hepatitis C-related liver disease each year [1]. Chronic HCV infection significantly impairs health-related quality of life (HRQoL), affecting mental, social, and physical function, as well as general health [2, 3]. Although often characterized as asymptomatic, 50–70% of individuals with chronic HCV infection report fatigue [4–6]. Furthermore, Sarkar et al. argue that treatment for HCV is warranted for patients without extensive liver damage if they are experiencing significant problems due to fatigue [7]. The potential causative factors of fatigue in HCV are unclear with some evidence suggesting fatigue results from comorbid depression and anxiety disorders, while other studies implicate the impact of HCV on liver fibrosis or neuroinvasion [7–11].

Over the past decade, the standard-of-care treatment for HCV infection has been a combination of peginterferon-α and ribavirin (PegIFN-α/RBV [PR]) [12, 13]. However, although sustained virologic response (SVR) rates have substantially improved with PR for some patients, a 48-week regimen of PR alone delivers suboptimal response rates (approximately 40–50%) in patients with HCV genotype 1 infection [12, 14, 15]. Furthermore, the incidence and severity of fatigue increases further during treatment with PR, which is attributed to multiple factors but most often to reductions in hemoglobin associated with treatment [16–18].

The introduction of direct-acting antiviral therapies such as NS3/4A protease inhibitors (PIs) in combination with PR heralds a new and promising advance for the treatment of HCV infection [19]. In addition to improved SVR rates compared with PR in both treatment-naïve and treatment-experienced patients with genotype 1 HCV infection, regimens containing a PI with PR allow clinicians to shorten PR treatment using response-guided therapy (RGT) [20, 21]. With RGT, treatment decisions are made according to whether patients achieve certain on-treatment virologic response milestones, thereby enabling patients to qualify for a shortened duration of PR therapy. More recently, promising results from two Phase II studies, PILLAR and ASPIRE, have been reported in treatment-naïve and treatment-experienced patients with genotype 1 HCV infection treated with triple therapy incorporating the investigational, once-daily, oral HCV NS3/4A PI, simeprevir (TMC435) [22, 23].

The aim of the current analysis was to examine the impact of adding simeprevir to PR therapy on patient-reported fatigue and health status in patients with chronic HCV infection enrolled in the PILLAR and ASPIRE studies, using the Fatigue Severity Scale (FSS) and the European Quality of Life 5-Dimensions (EQ-5D) questionnaires.

## Methods

### Study design

**Figure 1:**
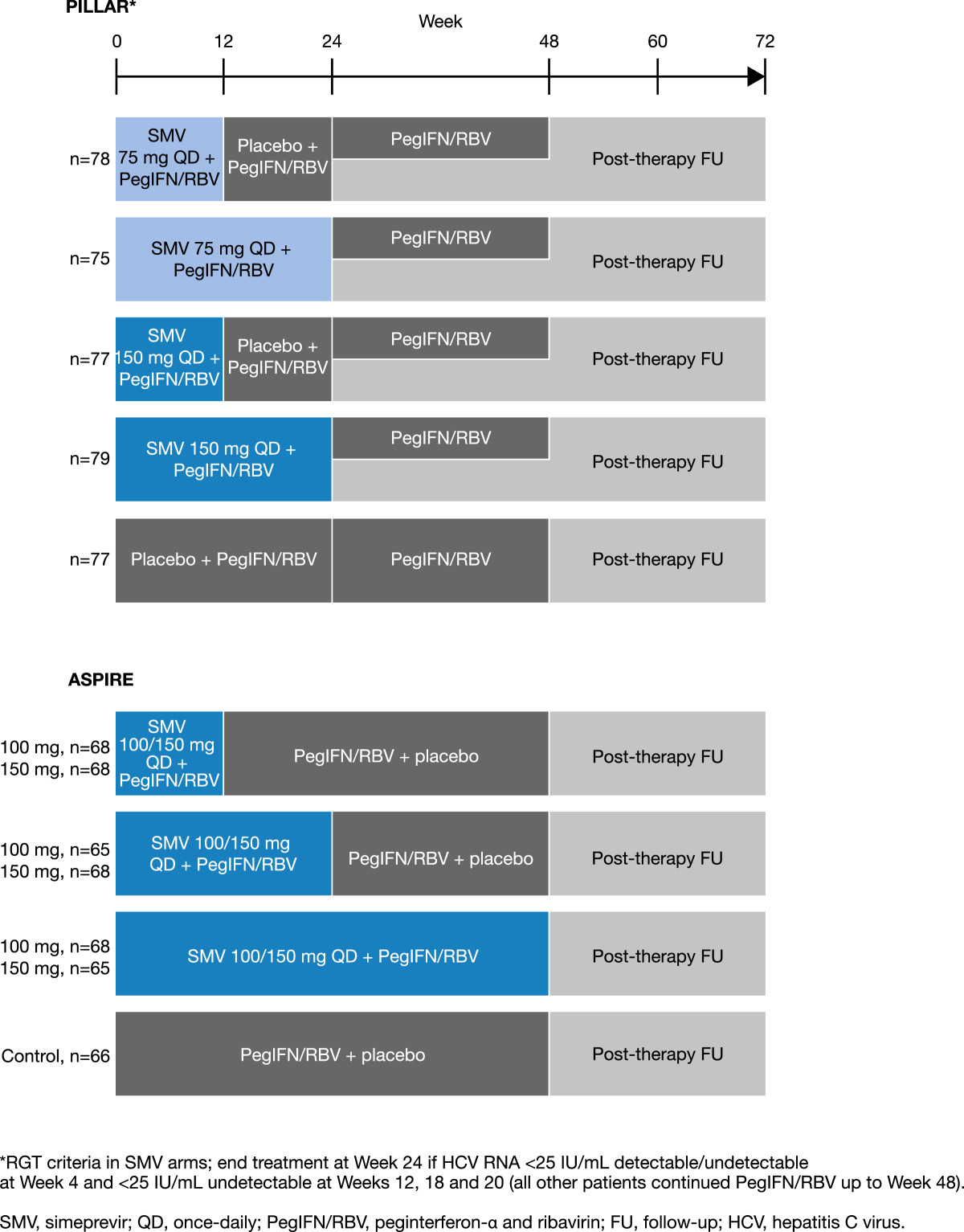
**PILLAR and ASPIRE trial designs.**

The PILLAR and ASPIRE trials were Phase IIb, multicenter, randomized, double-blind, placebo-controlled studies evaluating the efficacy and safety of once-daily simeprevir combined with PR in chronically infected treatment-naïve (PILLAR) or treatment-experienced (ASPIRE) HCV genotype 1 patients. The PILLAR and ASPIRE study designs are described in detail elsewhere [22, 23], therefore only a brief summary is provided here.In the PILLAR study treatment-naïve patients were randomized to one of five treatment regimens comprising simeprevir 75 or 150 mg administered orally once-daily in combination with PR (simeprevir/PR) or placebo in combination with PR (placebo/PR) (Figure 1). The treatment duration for triple therapy was either 12 or 24 weeks, with patients who were randomized to 12 weeks of triple therapy receiving an additional 12 weeks of PR. In addition, patients receiving regimens containing simeprevir were eligible for shortened duration of treatment using an RGT algorithm. On this basis, patients completed all therapy at Week 24 of treatment if they achieved HCV RNA <25 IU/mL at Week 4 and were undetectable at Weeks 12, 16, and 20. Simeprevir-treated participants who did not meet these virologic milestones continued therapy for up to 48 weeks, as did the PR group.

In the ASPIRE study, patients with null response, partial response, or relapse to prior PR received treatment comprising simeprevir 100 or 150 mg once daily for 12, 24, or 48 weeks plus PR for 48 weeks. In all of the simeprevir treatment arms, when patients were not receiving simeprevir, they received a matched placebo. For all patients, the 48-week treatment period was followed by post-treatment follow-up for up to 72 weeks after the start of treatment. No RGT was used in the ASPIRE study.

In all treatment groups in both studies, patients received PegIFN-α-2a administered as a subcutaneous injection (180 μg once weekly) and RBV as oral tablets (1000 or 1200 mg/day, dependent on body weight). Both study protocols were reviewed by the appropriate Independent Ethics Committee/Institutional Review Board at each participating study center and the studies were conducted in accordance with the ethical principles of the Declaration of Helsinki and in accordance with Good Clinical Practice guidelines. Between 30 March and 28 September 2009, 57 Institutional Ethics Committees in 15 countries approved the protocol for PILLAR. The protocol for ASPIRE was approved by 54 Institutional Ethics Committees in 14 countries between 17 September and 24 December 2009. A list of the investigators and Ethics Committees who approved these protocols is provided in the Additional file 1 for this article. All patients were required to provide written informed consent prior to participation in the studies.

In the PILLAR study, the primary endpoint was SVR at Week 72 (defined as the proportion of participants with HCV RNA <25 IU/mL and undetectable at Study Week 72); secondary endpoints included SVR at 12 and 24 weeks post-treatment. In the ASPIRE study the primary efficacy endpoint was the proportion of patients with an SVR defined as HCV RNA <25 IU/mL undetectable at 24 weeks after the planned end of treatment (SVR24). Secondary efficacy endpoints included the proportion of patients with a rapid virologic response (RVR; HCV RNA <25 IU/mL undetectable at Week 4) and proportion of patients with SVR 12 weeks after the planned end of treatment (SVR12).

### Patient-reported outcomes

Patients independently completed the 9-item FSS questionnaire [24, 25] and EQ-5D at baseline, throughout the PILLAR and ASPIRE trials (FSS: Weeks 4, 12, 24, 36, 48, 60, and 72; EQ-5D: Weeks 24, 48, and 72) and at the time of early treatment discontinuation, if applicable.

In common with the EQ-5D, the FSS is not specific for patients with HCV infection: both have been used across a wide range of chronic diseases [26–29]. The FSS has, however, been previously validated in patients with chronic hepatitis C [30–32]. In addition, it has been used to evaluate the experience of fatigue as a symptom of hepatitis C disease as well as a treatment adverse event (AE), where lower levels of fatigue were reported post-treatment in patients with SVR, and worsening fatigue was associated with treatment discontinuation [33].

Using the FSS questionnaire, patients were asked to assign a score of between 1 (completely disagree) and 7 (completely agree) to each of the 9 FSS items designed to rate the extent of fatigue symptoms and their impact on patient functioning (including motivation, exercise, physical function, carrying out duties, and interference with work, family, or social life); a higher score indicated a higher degree of fatigue. An FSS total score was then calculated based on the mean of the 9-item scores (range 1–7, higher total score indicates greater fatigue). The FSS in PILLAR used a 14-day recall period, while FSS recall in ASPIRE was ‘the past 7 days’.

The EQ-5D is a generic preference-based measure of HRQoL comprising five questions and a visual analog scale (VAS) to measure general health status [27, 34]. Patients rated their health states on five health domains: Mobility, Self Care, Usual Activities, Pain/Discomfort, and Anxiety/Depression. Domain scores for each dimension reflect one of three health states (level 1, no problems; level 2, some problems; level 3, extreme problems). The five domains were summarized using a descriptive system and as one single value, the EQ-5D valuation index (range 0–1). In order to determine the valuation index, all of the possible 243 unique health states that could be defined using this system were given a utility score using an existing tariff. The EQ-5D tariff represented the general public’s preferences as determined using time trade-off and differs according to country. For this study, the UK tariffs were used to assign population preference weights to the EQ-5D domains to produce the EQ-5D valuation index scores analyzed [35]. The EQ-5D VAS score utilized a score range from 0 to 100, with a score of 100 representing the best imaginable health status and a score of 0 the worst imaginable health status.

Both the FSS and EQ-5D questionnaires were only administered if a validated translation was available in the local language and only prior to all other trial-related procedures planned during the visit. To independently complete both questionnaires, patients had to be able to read and write and were not permitted to receive help from anyone accompanying them (e.g. family members and friends) or trial staff, in interpreting or responding to the questions. However, if a patient was unable to read, or had visual or other physical limitations that made it difficult to read or complete the questionnaires, trained trial staff could read the questions and response options aloud, and record the patient’s responses. Trial-site staff reviewed completed questionnaires to ensure that all questions were completed, and if missing items were noted, patients were asked to complete these items before any other study procedures were conducted.

### Data analysis

The 9-item responses in the FSS were combined into one total score per time point by calculating the mean of all non-missing items. If the number of missing items was 4 or more, the total score was defined as missing. Descriptive statistics of the actual FSS total scores and changes from baseline per time point were generated. The area under the curve (AUC) from baseline to Week 72 between the simeprevir/PR and the placebo/PR treatment groups was compared using a piecewise-linear mixed model. Treatment group and time were included as factors.

The incidence of level scores in each EQ-5D dimension (Mobility, Self-care, Usual Activities, Pain/Discomfort, and Anxiety/Depression), actual EQ-5D VAS scores (range 0–100) and EQ-5D index scores (range 0–1) and their changes from baseline were summarized per time point. Descriptive statistics of the actual values and the changes from baseline (for EQ-5D VAS and valuation index) were generated per time point. The utility decrement associated with both PR alone and simeprevir/PR therapy was calculated by comparing the baseline EQ-5D Index score with the average score captured during the year of treatment (Weeks 24–48). No statistical comparisons of EQ-5D scores were conducted as none were pre-specified in the *a priori* statistical analysis plan.

For both the FSS and EQ-5D, sensitivity analyses were performed based on the last-observation-carried-forward approach (LOCF).

The degree of change in FSS score that is considered clinically important in patients with HCV infection has not been reported in prior validation of the FSS [30]. Analyses to establish the amount of change in FSS scores that was clinically important for patients receiving treatment for HCV infection are included to help interpret trial results. A blinded analysis of FSS and EQ-5D data from PILLAR and ASPIRE were linked with spontaneous reports of fatigue AEs, encompassing the Medical Dictionary for Regulatory Activities (MedDRA) preferred terms ‘fatigue’ and ‘asthenia’, to the FSS scores. For each patient the first (and worst if more than one) fatigue AE occurring at any time within a recall period was retained until a worse AE was recorded during a later recall period. A case control method was then employed whereby a control was selected from among patients who did not experience a fatigue AE during the study by matching with gender, age and ethnicity. Independent analysis of FSS scores for both studies at baseline and at the visit at which the worst fatigue AE was recorded, and change from baseline FSS scores were compared for cases versus controls using t-tests.

## Results

### Patient characteristics

In total, 386 patients were randomized and treated in the PILLAR study (simeprevir/PR therapy, n = 309; placebo/PR, n = 77) and 462 patients were randomized and treated in the ASPIRE study (simeprevir/PR therapy, n = 396; placebo/PR, n = 66). Patient demographic and baseline disease characteristics for both studies are summarized in Table 1. The majority of patients were Caucasian and there was a slight predominance of males, particularly in the ASPIRE study. Median age overall (simeprevir/PR and placebo/PR groups combined) was 46.5 years (range 18–69) in the PILLAR study and 50.0 years (range 20–69) in the ASPIRE study. HCV RNA levels were >800 000 IU/mL in more than 80% of patients overall in both studies and over half of the patients had HCV genotype 1b infection (PILLAR, 54.3%; ASPIRE, 57.6%). Mean baseline FSS, EQ-5D VAS, and EQ-5D valuation index scores were comparable between the simeprevir/PR and placebo/PR treatment groups in both the PILLAR and ASPIRE studies and were also comparable between studies (Table 1).

**Table 1:** **Patient baseline demographic and disease characteristics for all treatment groups in PILLAR and ASPIRE**

	PILLAR		ASPIRE	
	All simeprevir (N = 309)	PR (N = 77)	All simeprevir (N = 396)	PR (N = 66)
Male, n (%)	174 (56.3)	39 (50.6)	269 (67.9)	42 (63.6)
Caucasian race, n (%)	288 (93.2)	74 (96.1)	366 (92.4)	62 (93.9)
Age (range), years^†^	47.0 (18–69)	45.0 (21–67)	50.0 (20–69)	50.5 (22–66)
Body mass index (range), kg/m^2 †^	24.9 (16.8–39.6)	25.6 (17.5–42.2)	27.0 (18.2–48.7)	28.0 (18.5–40.5)
HCV RNA (range) log_10_ IU/mL^†^	6.6 (3.5–8.1)	6.6 (4.3–7.5)	6.6 (3.5–7.7)	6.6 (5.2–7.6)
HCV RNA >800,000 IU/mL, n (%)	268 (86.7)	63 (81.8)	344 (86.9)	55 (83.3)
HCV genotype, N^‡^	307	76	389	66
1a, n (%)	144 (46.9)	29 (38.2)	161 (41.4)	27 (40.9)
1b, n (%)	161 (52.4)	47 (61.8)	223 (57.3)	39 (59.1)
Metavir score, N	309	77	391	64
F3, n (%)	46 (14.9)	7 (9.1)	73 (18.7)	13 (20.3)
F4, n (%)	1 (0.3)	0 (0)	73 (18.7)	10 (15.6)
EQ-5D, N	305	77	384	64
Baseline valuation index, mean (SE)	0.9 (0.01)	0.9 (0.03)	0.9 (0.01)	0.9 (0.02)
Baseline VAS, mean (SE)	82.2 (0.91)	83.6 (1.42)^§^	80.3 (0.83)	80.5 (2.22)
FSS, N	195	50	385	64
Baseline, mean (SE)	3.3 (0.12)	3.2 (0.21)	3.4 (0.08)	3.2 (0.22)

^†^Median; ^‡^NS5B sequence-based assay; ^§^n = 76.

*PR* placebo/peginterferon-α and ribavirin, *HCV* hepatitis C virus, *EQ-5D* European Quality of Life 5-Dimensions, *SE* standard error, *FSS* Fatigue Severity Scale, *VAS* visual analog scale.

### Clinical efficacy

**Figure 2:**
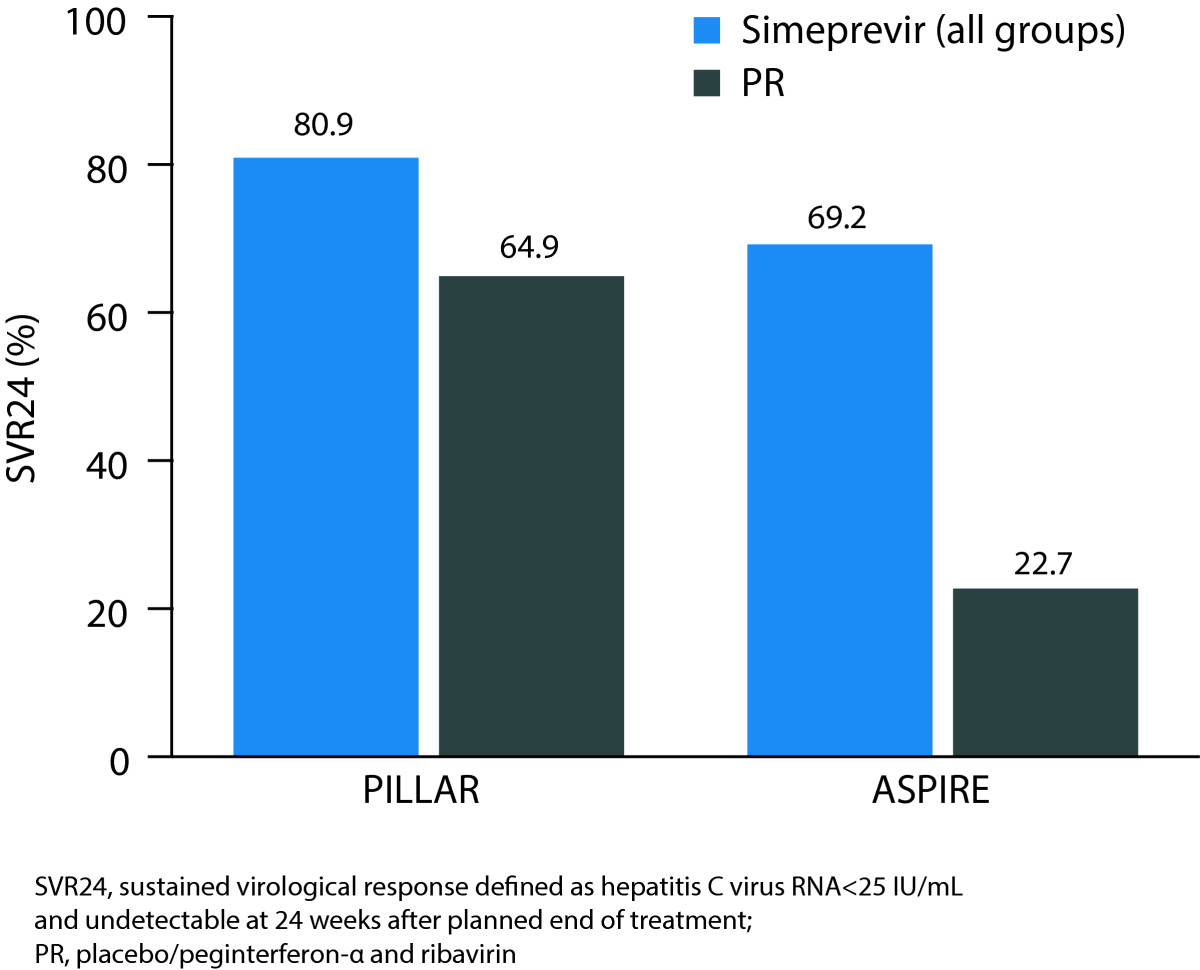
**Proportion of simeprevir/PR-treated patients achieving SVR24 in the PILLAR and ASPIRE studies.**

Clinical efficacy data from the PILLAR and ASPIRE studies have been published elsewhere [22, 23]. In the PILLAR study a higher proportion of patients achieved the primary endpoint of SVR72 (78.6% simeprevir/PR vs 64.9% of placebo/PR patients). Furthermore, among the simeprevir/PR-treated patients in the PILLAR study, 73.5% achieved RVR and 92.1% of these patients subsequently achieved SVR24. In comparison, only 5.2% of patients in the placebo/PR group achieved RVR. Overall, 82.2% of simeprevir/PR-treated patients completed treatment by Week 24 according to RGT criteria (HCV RNA <25 IU/mL at Week 4, then undetectable at Weeks 12, 16, 20) and 91.3% of these patients subsequently achieved SVR24. Similarly, in the ASPIRE study, a higher proportion of simeprevir/PR-treated patients compared with placebo/PR-treated patients achieved SVR24 (primary endpoint, 69.2 vs 22.7%) and RVR (62.4 vs 1.5%) (Figure 2).

### Fatigue as an AE

Fatigue was reported as an AE in 42.4% of all simeprevir/PR-treated patients in the PILLAR study (vs 48.1% of placebo/PR-treated patients) and 22.3% of these patients had a fatigue AE that was considered by the investigator to possibly be related to the study drug (vs 23.4% of placebo/PR-treated patients). A similar incidence of fatigue was documented in the ASPIRE study: 43.9% of simeprevir/PR-treated patients experienced a fatigue AE (vs 43.9% of placebo/PR-treated patients) and 24.2% of these patients had a fatigue AE that was considered to possibly be related to the study drug (vs 22.7% of placebo/PR-treated patients). The incidence of Grade 3/4 fatigue among simeprevir/PR-treated patients was <1.0% in both studies (Grade 3/4 defined according to the World Health Organization toxicity grading system as severe with marked activity limitation, or potentially life-threatening with extreme activity limitation).

### FSS

A total of 245 (63.5%) patients in the PILLAR study and 449 (97.2%) patients in the ASPIRE study completed the FSS during the baseline visit.^a^ Mean baseline FSS scores in the simeprevir/PR and placebo/PR groups were comparable within and between studies (all simeprevir/PR groups: 3.31 [PILLAR], 3.37 [ASPIRE]; Table 2) and approximately 1 point higher than the established normative value for FSS scores in healthy adults (mean ± standard error [SE]: 2.3 ± 0.7) but roughly one point lower than patients with multiple sclerosis (MS) or systemic lupus erythematosis (SLE) [26].

**Table 2:** **Mean change from baseline in FSS total scores at EOT and EOF**

FSS total score	PILLAR, n (%)		ASPIRE, n (%)	
	All simeprevir (n = 309)	PR (n = 77)	All simeprevir (n = 396)	PR (n = 66)
Week 24, N	174 (EOT)^†^	48	343	52
Mean change (±SE)	1.16 (0.120)	1.25 (0.237)	0.99 (0.080)	0.73 (0.217)
Week 36, N	172	45	346	53
Mean change (±SE)	0.06 (0.130)	1.08 (0.272)	0.91 (0.086)	0.43 (0.221)
Week 48, N	166	43 (EOT)	344 (EOT)	52 (EOT)
Mean change (±SE)	−0.19 (0.132)	1.19 (0.267)	0.85 (0.085)	0.50 (0.220)
Week 60, N	164	43	342	54
Mean change (±SE)	−0.49 (0.112)	−0.15 (0.186)	−0.05 (0.079)	−0.03 (0.188)
Week 72, N	164 (EOF)	44 (EOF)	338 (EOF)	56 (EOF)
Mean change (±SE)	−0.50 (0.108)	−0.40 (0.219)	−0.22 (0.079)	−0.18 (0.202)

^†^For the majority of SMV-treated patients, EOT was 24 weeks; 55 patients in the SMV group continued PR therapy through to Week 48 as they did not meet the response-guided therapy criteria at Week 24. *FSS* Fatigue Severity Scale, *EOT* end of treatment, *EOF* end of follow-up, *PR* placebo/peginterferon-α and ribavirin, *SE* standard error.

**Figure 3:**
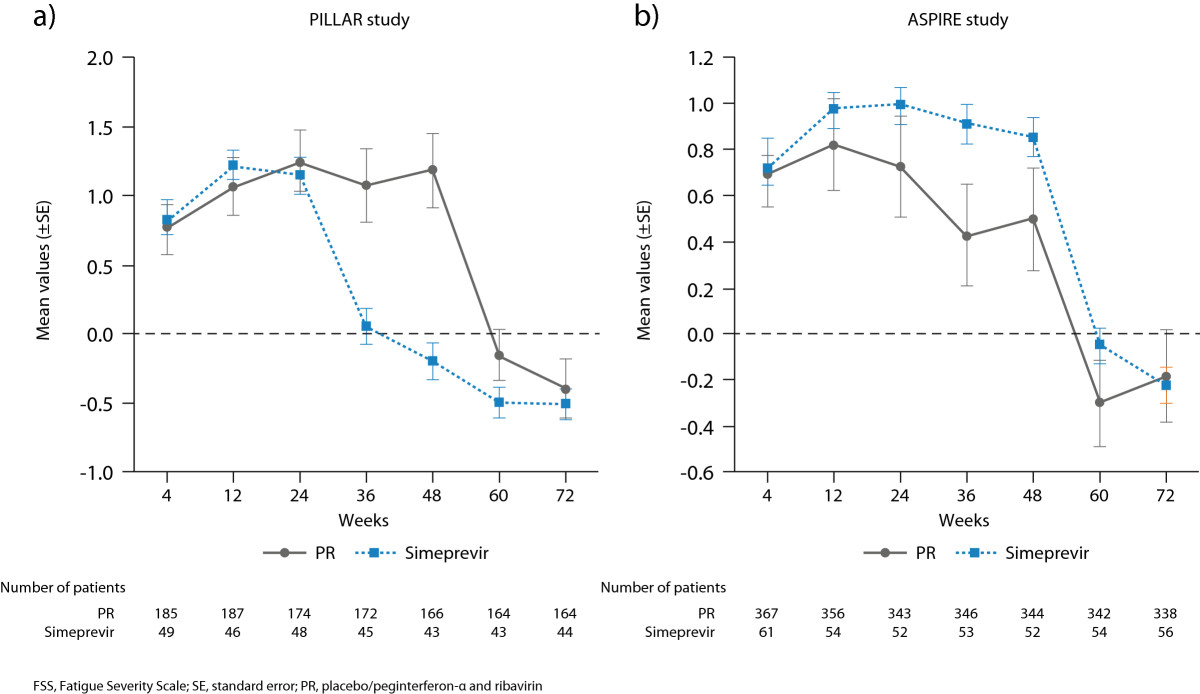
**Change from baseline in FSS total score up to Week 72 for all simeprevir/PR treatment groups combined vs placebo/PR in a) the PILLAR study and b) the ASPIRE study.**

**Figure 4:**
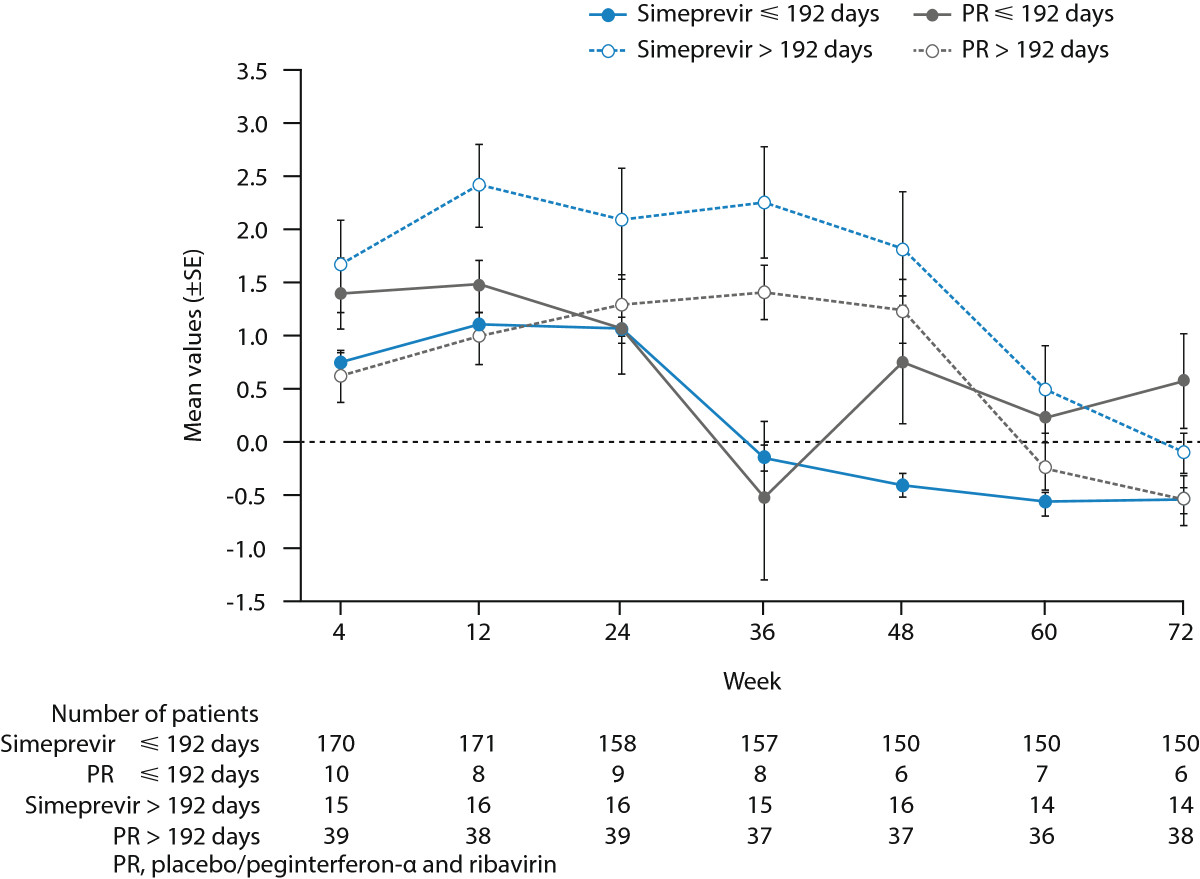
**Mean change (±SE) from baseline in FSS total score up to Week 72 for all simeprevir/PR treatment groups combined vs placebo/PR according to treatment duration in the PILLAR study.**

During the first 24 weeks of treatment, mean FSS total scores were generally comparable and increased compared with baseline among simeprevir/PR- and placebo/PR-treated patients in both studies indicating a higher degree of fatigue. After 24 weeks of treatment in the PILLAR study, an earlier decline in mean FSS total score was observed in the simeprevir/PR group compared to the placebo/PR group. Specifically, by Week 36 in the PILLAR study (12 weeks after most patients [82.2%] completed treatment), mean FSS total score had decreased to a value comparable to baseline (mean change vs baseline 0.06) among the simeprevir/PR-treated patients and this decreased further to below the baseline value at Week 48 and beyond (Table 2; Figure 3). In comparison, in the PR group, improvement in FSS total score to a value comparable to or below the baseline value was not evident until Week 60. Figure 4 compares change in total FSS score from baseline over time for each treatment group stratified by duration of treatment (≤192 days vs >192 days) for the PILLAR study. In the simeprevir/PR treatment group, the best outcome in terms of reduction in fatigue was achieved by those patients who met RGT criteria and were able to complete the optimum duration of simeprevir/PR therapy (24 weeks).

In the ASPIRE study, improvement in fatigue comparable to baseline levels was reported at Week 60 in both the simeprevir/PR and placebo/PR groups. The earlier improvement in fatigue in the PILLAR study (Week 36) compared with the ASPIRE study (Week 60) was consistent with the implementation of RGT in PILLAR (but not ASPIRE) allowing the PILLAR study patients to qualify for shortened treatment duration and to discontinue therapy at Week 24.

Statistical analyses of the PILLAR study using AUC indicated significantly (*P* < 0.001) less fatigue over the 72-week treatment and follow-up period for patients treated with simeprevir/PR (AUC = 250.2 [SE = 5.0]) than with placebo/PR (AUC = 283.9 [SE = 8.8]). In the ASPIRE study, AUC indicated lower fatigue overall in the placebo/PR group (AUC = 268.9 [SE = 9.4]) compared to the simeprevir/PR group (AUC = 287.3 [SE = 5.1]) although this difference was not statistically significant.

Sensitivity analyses in both studies, including a LOCF approach, and comparison of characteristics of patients with and without baseline FSS data in the PILLAR study (Table 3), indicated that the FSS results were unaffected by missing data.

**Table 3:** **Baseline demographic and disease characteristics of patients in PILLAR with and without FSS scores at baseline**

	With baseline FSS		Without baseline FSS	
	All simeprevir (N = 195)	PR (N = 50)	All simeprevir (N = 114)	PR (N = 27)
Male, n (%)	115 (59.0)	23 (46.0)	59 (51.8)	16 (59.3)
Caucasian race, n (%)	180 (92.3)	49 (98.0)	108 (94.7)	25 (92.6)
Age (range), years^†^	48.0 (18–69)	47.5 (21–61)	42.5 (18–66)	42.0 (21–67)
Body mass index (range), kg/m^2†^	25.1 (17.1–38.2)	25.7 (17.6–36.0)	24.7 (16.8–39.6)	25.6 (17.5–42.2)
HCV RNA (range) log_10_ IU/mL^†^	6.6 (3.5–8.1)	6.6 (4.3–7.5)	6.5 (5.1–7.5)	6.2 (5.4–7.1)
HCV RNA >800,000 IU/mL, n (%)	170 (87.2)	43 (86.0)	98 (86.0)	20 (74.1)
HCV genotype, N^‡^				
1a, n (%)	105 (53.8)	21 (42.0)	40 (35.1)	9 (33.3)
1b, n (%)	88 (45.1)	29 (58.0)	73 (64.0)	18 (66.7)
Metavir score, N				
F3, n (%)	33 (16.9)	5 (10.0)	13 (11.4)	2 (7.4)
F4, n (%)	0 (0.0)	0 (0.0)	1 (0.9)	0 (0.0)

^†^Median; ^‡^NS5B sequence-based assay.

*PR* placebo/peginterferon-α and ribavirin, *HCV* hepatitis C virus, *FSS* Fatigue Severity Scale.

Correspondence of FSS self-reported fatigue severity with fatigue AE data was comparable between the two trials. The difference between the mean change from baseline in FSS total scores for patients with fatigue AEs compared with matched controls, who did not experience a fatigue AE, was 0.6 in the PILLAR study (mean FSS change 1.1 vs 0.5 for controls; *P* = 0.011) and 0.5 in the ASPIRE study (mean FSS change 0.9 vs 0.4 for controls; *P* < 0.001).

### EQ-5D descriptive system

At baseline, the proportion of patients reporting extreme problems with their health status (most severe category) in the simeprevir/PR and placebo/PR groups was low in all dimensions (<4% of patients in any dimension, in both studies). During both the PILLAR and ASPIRE studies there were generally fewer problems in terms of Mobility or Self-care. Pain, Mood Disturbances, and impairment in Usual Activities were generally the most common domains affected during treatment (Table 4).

**Table 4:** **Summary of patients reporting any health problem by EQ-5D domain in the PILLAR and ASPIRE studies**

EQ-5D domain	Study visit	PILLAR, n/N (%)		ASPIRE, n/N (%)	
		All simeprevir	Placebo/PR	All simeprevir	Placebo/PR
Mobiity	Baseline	21/306 (6.9)	5/77 (6.5)	27/386 (7.0)	6/64 (9.4)
	Week 24	61/285 (21.5)	6/72 (8.3)	80/355 (22.6)	14/51 (27.5)
	Week 48	25/271 (9.3)	11/67 (16.4)	76/352 (21.6)	11/55 (20.0)
	Week 72	16/279 (5.7)	5/68 (7.4)	38/340 (11.2)	11/55 (20.0)
Self-care	Baseline	3/306 (1.0)	2/77 (2.6)	5/387 (1.3)	2/64 (3.1)
	Week 24	6/285 (2.1)	2/72 (2.8)	13/354 (3.7)	3/51 (5.9)
	Week 48	5/271 (1.9)	2/68 (2.9)	18/352 (5.1)	5/55 (9.1)
	Week 72	6/279 (2.2)	1/68 (1.5)	9/339 (2.7)	3/55 (5.5)
Usual activities	Baseline	32/305 (10.5)	4/77 (5.2)	49/387 (12.6)	9/64 (14.1)
	Week 24	148/284 (52.1)	36/72 (50.0)	159/354 (44.9)	18/51 (35.3)
	Week 48	46/271 (17.0)	31/68 (45.5)	153/351 (43.6)	17/55 (30.9)
	Week 72	36/279 (12.9)	8/68 (11.8)	55/338 (16.3)	12/55 (21.8)
Pain/discomfort	Baseline	83/306 (27.1)	17/77 (22.1)	109/385 (28.3)	15/64 (23.4)
	Week 24	154/285 (54.0)	45/72 (62.5)	193/354 (54.5)	21/51 (41.2)
	Week 48	73/271 (27.0)	38/68 (55.9)	188/349 (53.9)	22/55 (40.0)
	Week 72	64/279 (23.0)	19/68 (27.9)	107/337 (31.8)	21/55 (38.2)
Anxiety/	Baseline	71/306 (23.2)	15/77 (19.5)	80/385 (20.8)	11/64 (17.2)
depression	Week 24	130/284 (45.7)	39/72 (54.2)	165/355 (46.4)	21/51 (41.2)
	Week 48	65/271 (23.9)	24/68 (35.3)	168/351 (47.9)	20/55 (36.4)
	Week 72	63/279 (22.6)	15/68 (22.0)	94/338 (27.9)	18/55 (32.7)

*EQ-5D* European Quality of Life 5-Dimensions, *PR* placebo/peginterferon-α and ribavirin.

In both trials, EQ-5D was only collected at baseline, Week 24, Week 48, and Week 72. In the treatment-naïve PILLAR study population, the proportion of simeprevir/PR- and placebo/PR-treated patients with problems relating to Usual Activities, Pain, and Anxiety/Depression and to a lesser extent Mobility increased at Week 24 relative to baseline. By Week 48 (24 weeks after most patients [82.2%] completed treatment) in the simeprevir/PR groups, the number of patients with problems in these dimensions had decreased compared with Week 24. A similar improvement (Week 48 vs Week 24) was evident in the placebo/PR group for the Anxiety/Depression domain, although the proportion of patients with problems was higher than in the simeprevir/PR group (Table 4).

Similar to the PILLAR study, problems in the Pain/Discomfort, Usual Activities, Anxiety/Depression, and to a lesser extent Mobility domains increased at Week 24 versus baseline among simeprevir/PR- and placebo/PR-treated patients enrolled in the ASPIRE study. However, in contrast, the ASPIRE patient population, who were all prior HCV treatment-experienced patients (patients with null or partial response or who relapsed), did not experience a reduction in health status problems (any dimension) at Week 48 versus Week 24; overall, health status problems were generally similar across the simeprevir/PR and placebo/PR treatment groups at these timepoints. By Week 72, the proportion of patients with health status problems (any dimension) had returned to levels similar to baseline in both studies.

### EQ-5D valuation index

**Figure 5:**
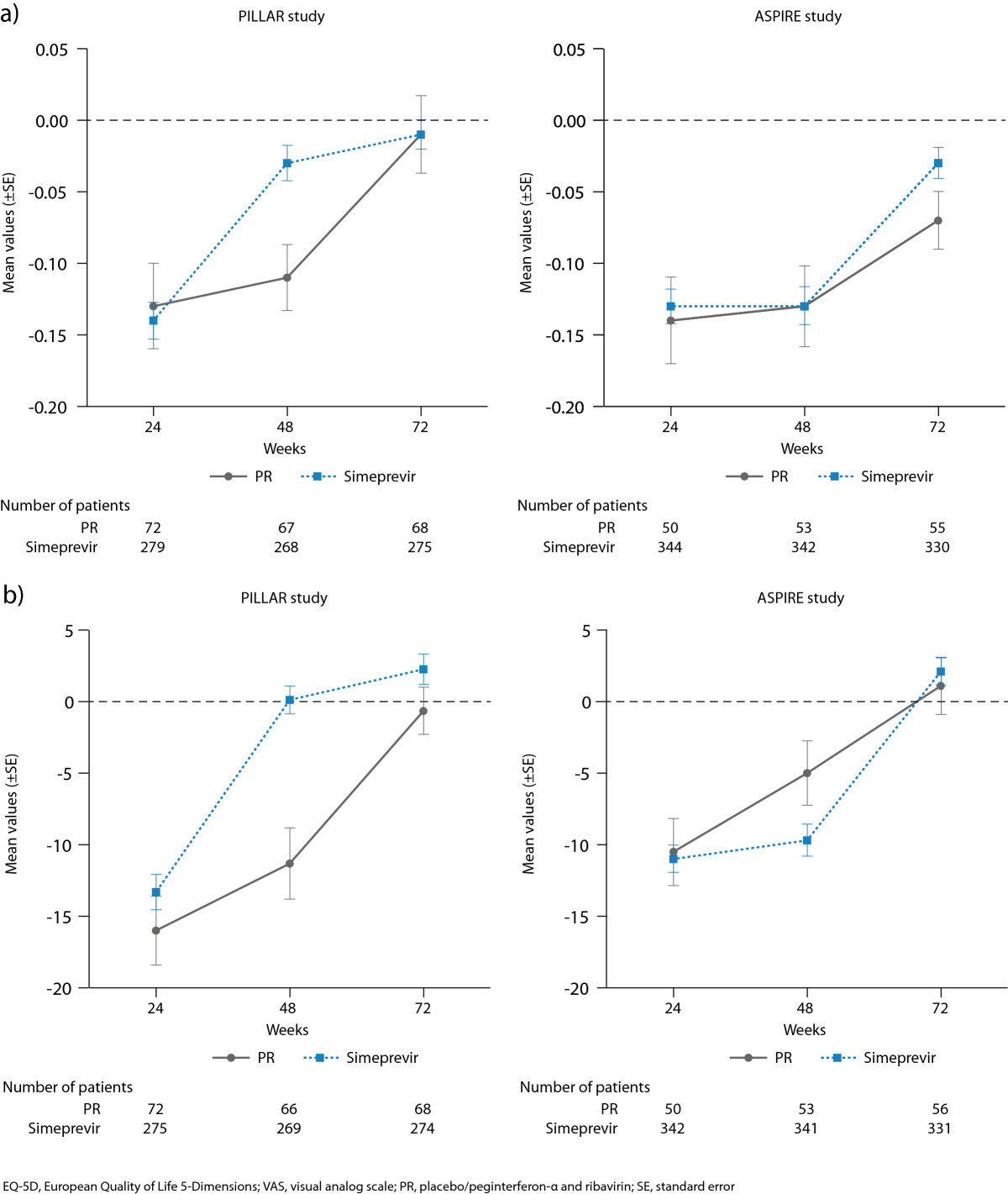
**Mean change from baseline in a) EQ-5D valuation index values and b) EQ-5D VAS for all simeprevir/PR treatment groups combined vs placebo/PR in the PILLAR and ASPIRE studies.**

Mean EQ-5D valuation index values decreased compared with baseline at Week 24 (indicating worsening of health status) in the simeprevir/PR and placebo/PR groups in both studies (PILLAR: −0.14, −0.13; ASPIRE: −0.13, −0.14, respectively) with no relevant differences between treatment groups (Figure 5). At Week 48 in the PILLAR study, the mean EQ-5D valuation index was increased compared with Week 24 in the simeprevir/PR group (mean change from baseline: −0.03), indicating health status improvement while in the placebo/PR group the mean decrease from baseline remained relatively stable at Week 48 compared with Week 24 (−0.11). The mean decrement in utility during Weeks 0–48 was smaller among the simeprevir/PR-treated patients (−0.067; 95% confidence interval [CI]: −0.111, −0.024) compared with the placebo/PR-treated patients (−0.119; 95% CI: −0.164, −0.075), representing a difference of 0.052 (95% CI: −0.011, 0.114). In contrast, no improvement in health status was observed in the ASPIRE study at Week 48 in either the simeprevir/PR or placebo/PR group (mean change from baseline: −0.13, −0.13, respectively). The mean decrement in utility during Weeks 0–48 was smaller among the simeprevir/PR-treated patients (−0.135; 95% CI: −0.187, −0.082) than among placebo/PR-treated patients (−0.155; 95% CI: −0.215, −0.095) representing a difference of 0.02024 (−0.0598 to 0.10028). By Week 72, mean EQ-5D valuation index values had increased to values similar to those reported at baseline in both the simeprevir/PR and placebo/PR treatment groups in both studies (Figure 5).

### EQ-5D VAS

After 24 weeks of treatment in the PILLAR and ASPIRE studies, both simeprevir/PR- and placebo/PR-treated patients rated their health status as having significantly worsened, as denoted by a decrease in mean VAS values compared with baseline of more than 10 points (PILLAR: −13.3, −16.0; ASPIRE: −11.0, −10.5, respectively) (Figure 5). In the PILLAR study, mean VAS returned to values similar to baseline by Week 48 (mean VAS change 0.12) and was improved versus baseline by Week 72 (2.26) in the simeprevir/PR group. In the ASPIRE study mean VAS values were worse versus baseline at Week 48 but had improved versus baseline by Week 72 (mean VAS change −9.7 and 2.1, respectively).

**Figure 6:**
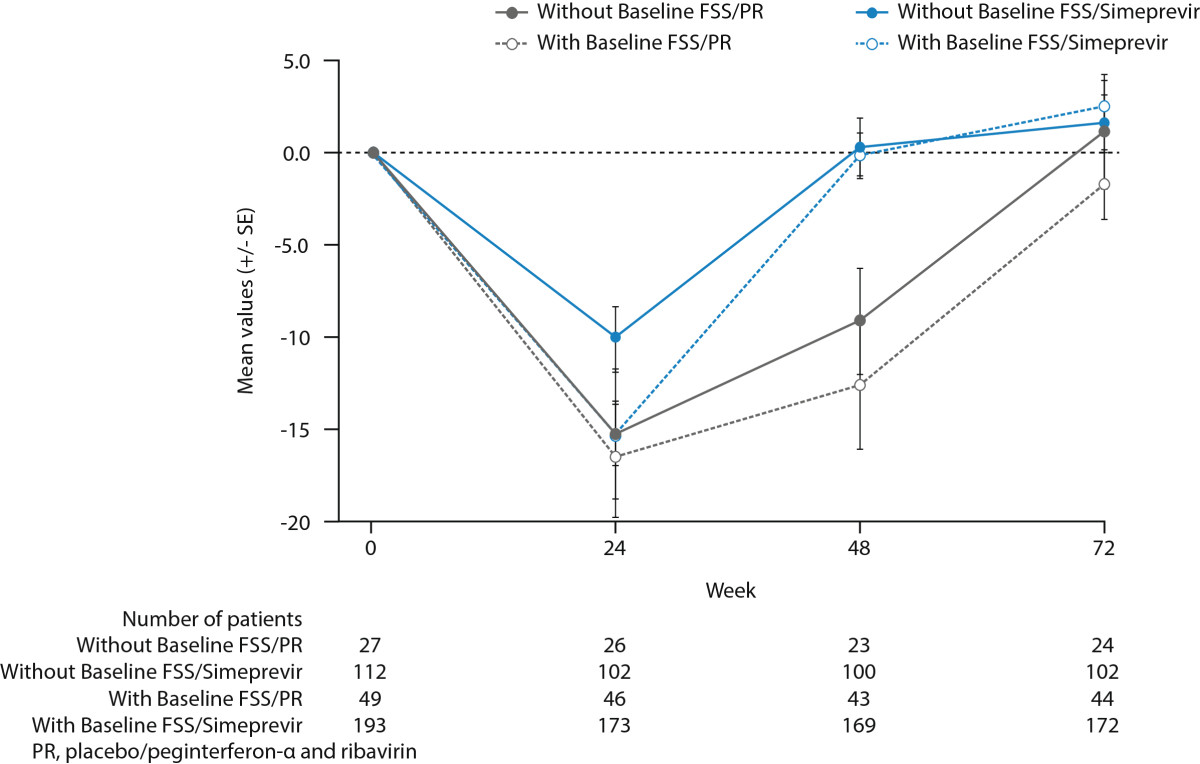
**Mean EQ-5D (±SE) changes over time in the Visual Analogue Scale (VAS) by treatment group for patients with and without FSS scores at baseline.**

Mean EQ-5D VAS scores in the simeprevir/PR group remained lower at Week 24 and 48 than in the placebo/PR group in the ASPIRE study. This is likely a reflection that more patients in the simeprevir/PR group continued on treatment with PR through Week 48 while the placebo/PR-treated patients were more likely to discontinue treatment by Week 20 due to meeting a virology stopping rule that indicated treatment failure. In the placebo/PR groups, mean EQ-5D VAS did not return to values similar to baseline until Week 72 in either study.Sensitivity analyses in each study, including a LOCF approach, and comparison of patients with and without baseline FSS scores in the PILLAR study (Figure 6) indicated that the EQ-5D VAS results were unaffected by missing data.

## Discussion

In the current analysis we evaluated patient-reported fatigue and health status associated with the addition of simeprevir to PegIFN/RBV in treatment-naïve and treatment-experienced patients with HCV genotype 1 infection. Fatigue is the most common symptom reported by individuals infected with HCV and is one of the most common treatment-related AEs among those who undergo treatment [16–18]. Increased fatigue is associated with reductions in HRQoL during treatment. The findings in both treatment-naïve and treatment-experienced patients indicate that the addition of simeprevir to PegIFN/RBV does not worsen fatigue and other health-related outcomes, above that already observed with PegIFN/RBV alone. As expected in both the PILLAR and ASPIRE studies, fatigue and other health-related dimensions initially worsened during therapy in all treatment groups (simeprevir/PR and placebo/PR); however, during the second part of treatment (Week 24 and beyond) fatigue severity and health status tended to stabilize and did not worsen further. Among the EQ-5D health dimensions assessed, Usual Activities, Pain Discomfort, and Anxiety/Depression all initially worsened during HCV therapy whilst Mobility and, to a greater extent, Self-Care remained primarily unaffected by treatment. A lower on-treatment disutility observed in the simeprevir/PR group compared with the placebo/PR group indicates less time with worse health status versus baseline in the simeprevir/PR group.

In the PILLAR study, the shorter PR treatment duration and higher SVR rates in the simeprevir/PR groups enabled simeprevir/PR-treated patients to experience less time with fatigue and lower health functioning compared with placebo/PR-treated patients. Of particular note was the fact that the majority (82.2%) of treatment-naïve patients treated with simeprevir/PR in the PILLAR study stopped all therapy at Week 24 according to the criteria for RGT; termination of therapy at Week 24 was subsequently followed by improvements in FSS and EQ-5D scores by the next available timepoint, denoting a reduction in fatigue and improvement in health status, among simeprevir/PR-treated patients. For the small number of simeprevir/PR patients who achieved insufficient viral response to meet RGT (n = 15) and received a longer duration of PR therapy (>192 days) higher fatigue scores were reported up to Week 48. In addition, fatigue scores were also higher up to Week 48 for patients in the placebo/PR group who were able to remain on treatment through to this time point (i.e. did not discontinue placebo/PR therapy early due to AEs or lack of viral response). These findings underscore the benefit, in terms of patient-reported fatigue and perceived health status, of a shorter overall duration of treatment for chronic HCV infection, which can be realized for the majority of patients treated with simeprevir/PR through the implementation of RGT.

In ASPIRE, where patients had previously failed to sustain a viral response following treatment with interferon, treatment with PR was scheduled to last until Week 48 for all patients. However, patients in the placebo/PR group were more likely to discontinue all treatment by Week 20 because they were unable to achieve adequate virologic response to treatment. The shorter duration of PR treatment in the placebo/PR group coupled with no RGT permitted for the simeprevir/PR-treated patients led to better HRQoL (higher EQ-VD VAS scores) at Weeks 24 and 48 in the placebo/PR group compared to the simeprevir/PR group. Focusing on this temporary advantage in HRQoL in the placebo/PR group at Weeks 24 and 48 ignores the long-term HRQoL consequences of persistent HCV infection for these patients who failed to achieve SVR with PR treatment alone.

Patient-reported fatigue data provide an important addition to fatigue-related AE data documented during the course of a clinical trial. The incidence and severity of fatigue AEs were comparable in all treatment groups in both studies (42.4−48.1% of patients). However, patient reports enrich our understanding of the fatigue experienced by patients in each study. Adding simeprevir to PR did not increase the severity of fatigue in either study; however, when virologic response was used to guide the duration of PR therapy (PILLAR only), the duration of treatment-related increases in fatigue was significantly reduced. Used in conjunction with AE data, patient-reported data could be used to aid discussion between patients and clinicians regarding what to expect from treatment and may ultimately help to inform treatment decisions in the HCV treatment setting. Specifically, the data could enable clinicians to advise patients on how long they are likely to experience fatigue and which aspects of daily functioning are most likely to be affected and when. For example, our data suggest that patients are more likely to experience worsening of usual activities, pain and discomfort, and mood-related symptoms compared with mobility or inability to provide self-care during HCV therapy, and that these effects are likely to occur for as long as they are on PR therapy.

Given the high incidence of fatigue among HCV-infected patients treated with antiviral therapy there is a need for a short, simple, reliable, and valid instrument to assess fatigue in HCV and inform treatment decisions. The findings from a recent validation study suggest that the FSS offers a reliable assessment of self-reported fatigue among patients with chronic HCV infection [25]. An FSS score of 2.3 is the recommended normal reference value for general population fatigue levels [28]. In both the PILLAR and ASPIRE studies, the FSS mean scores at baseline for HCV-infected patients were in excess of 3.0 which suggests many patients had clinically important fatigue prior to initiating treatment. By comparison, mean FSS scores indicating severe fatigue have been reported using the FSS in patients with MS (4.66) and SLE (4.7) [26]. Given FSS scores in all treatment groups in both studies increased to levels similar to those reported by patients with MS or SLE while patients were taking PR, there is clear evidence that shortening the duration of fatigue associated with HCV treatment is a significant clinical benefit for patients.

The blinded analyses from PILLAR and ASPIRE linking fatigue AEs to FSS scores also support the validity of the FSS as a measure of fatigue in HCV and indicate that mean changes in FSS scores as small as 0.5 are clinically important for patients with chronic HCV infection. A recent validation of the FSS based on data from PILLAR and ASPIRE also concluded that an interpretable and meaningful improvement in fatigue occurs when there is an observed-group mean change in FSS total score of between 0.33 and 0.82 [25]. Phase III simeprevir studies using the FSS evaluated response to treatment using these criteria and found shorter duration of worsening in fatigue associated with simeprevir/PR treatment than with placebo/PR in treatment-naïve patients and in patients who relapsed following PR treatment [36]. However, as the response criteria were created based on the PILLAR and ASPIRE data, a responder analysis was not pre-specified or conducted for the Phase IIb PILLAR and ASPIRE trials.

The current analysis was subject to some study limitations. In the PILLAR study, patients completed an FSS with a 14-day (2-week) recall while patients in the ASPIRE study completed the FSS using a 7-day (1-week) recall of symptoms and functioning problems. It is not possible to identify how much the difference in recall period may have affected FSS scores in this study. However, the chronicity of fatigue in this population and the close association between fatigue scores and PR treatment observed in this and other trials [36] suggests that the recall made little difference in this chronically ill patient population who were on fatigue-inducing treatments for many weeks.

In both the PILLAR and ASPIRE studies, patients and clinicians were blinded to the laboratory HCV RNA results and to why treatment was stopped prior to Week 48. Treatment could be stopped for several reasons: due to treatment failure (lack of response), severe AEs, or in PILLAR due to virologic response at Weeks 4 and 12. Sensitivity analyses compared FSS scores for patients treated with simeprevir/PR who had 192 days of treatment or less with those who completed more than 192 days of treatment (24 weeks) to evaluate the impact of RGT on FSS scores. Regardless of why patients discontinued treatment (because of meeting a stopping rule, meeting RGT criteria, or completing 48 weeks of treatment), the patient-reported outcome data were consistent with these problems being determined primarily by duration of PegIFN/RBV treatment.

It is important to recognize that patients in both the PILLAR and ASPIRE studies were blinded to simeprevir treatment assignment; however, PR treatment could not be blinded when treatment was stopped, although the reason for stopping was not disclosed. The strong association seen between duration of PR and patient-reported outcomes may be, in part, due to the inability to blind subjects to PR treatment duration.

This study was also limited by the fact that approximately 40% of patients in the PILLAR study did not have baseline FSS data either because translations of the FSS questionnaire in the patients’ native language were not available when the first study sites began patient enrolment or because of an error in the response scale for the FSS completed by the patients who initially entered the trial. Post hoc comparisons of patient demographics, disease characteristics at baseline, and EQ-5D VAS over the course of the study for patients with and without FSS at baseline indicated no major differences between the patients without baseline FSS and those patients included in the FSS analysis (Table 3, Figure 6). These sensitivity analyses found no evidence to suggest that the missing FSS data biased the results.

Both studies used procedures consistent with recommended practice to reduce missing data in patient questionnaires completed during clinical trials. The requirement that staff check each questionnaire a patient completed and ask that all questions be answered may have introduced bias in these results that cannot be evaluated here. Future studies that ask patients to complete questionnaires on computerized devices that can remind patients to answer any skipped questions will help eliminate any bias that may be introduced by asking staff to check for missing data that are required for paper- and pencil-administration.

## Conclusions

Patients in the PILLAR and ASPIRE trials with chronic HCV infection treated with simeprevir in combination with PR experience a similar safety/tolerability profile and a higher SVR rate compared with patients treated with PR alone. For treatment-naïve patients in the PILLAR study, high SVR rates were achieved even though most patients who received simeprevir were able to limit PR treatment to only 24 weeks because they met RGT criteria. The clinical benefits associated with the addition of simeprevir arise without a significant increase in patient-reported fatigue beyond that reported in patients treated with PR alone, and for many patients is associated with shorter duration of health impairment (fatigue and health status) compared with PR.

## Endnote

^a^The PILLAR study was the first simeprevir trial to include patient-reported outcome endpoints. Sites that enrolled the first patients did not have appropriate language translations of the patient-reported outcome instruments for their subjects to complete at their baseline visit. Some of these patients subsequently completed patient-reported outcome instruments at post-baseline visits but data from these patients were not included in the treatment comparisons due to lack of baseline data.

## Authors’ information

JS is Director Patient Reported Outcomes responsible for patient self-report assessments in clinical development studies conducted by Janssen’s Global Infectious Disease and Vaccines therapeutic area. KR is a psychometrician with a PhD in Quantitative Psychology from UNC Chapel Hill; she has worked in the planning and analysis of clinical trials for over 15 years, with a specialization in developing, validating and implementing patient-reported outcomes for clinical trial endpoints. MF is an Associate Director within Clinical Biostatistics at Janssen R&D, US. KC is a Visiting Researcher at the London School of Economics and Political Sciences. MBM is the Medical Lead of the TMC435 (simeprevir) program at Janssen.

## Electronic supplementary material

Additional file 1: Ethics Review Committees providing approval for PILLAR and ASPIRE.(PDF 129 KB)

Below are the links to the authors’ original submitted files for images.

Authors’ original file for figure 1

Authors’ original file for figure 2

Authors’ original file for figure 3

Authors’ original file for figure 4

Authors’ original file for figure 5

Authors’ original file for figure 6

Authors’ original file for figure 7

## References

[1] World Health Organization: 63rd World Health Assembly. Provisional agenda item 11_12; 25 March 2010. http://apps.who.int/gb/ebwha/pdf_files/WHA63/A63_15-en.pdf,

[2] Conrad S, Garrett LE, Cooksley WG, Dunne MP, MacDonald GA (2006). Living with chronic hepatitis C means ‘you just haven’t got a normal life any more’. Chronic Illn.

[3] Groessl EJ, Weingart KR, Kaplan RM, Clark JA, Gifford AL, Ho SB (2008). Living with hepatitis C: qualitative interviews with hepatitis C-infected veterans. J Gen Intern Med.

[4] Hassoun Z, Willems B, Deslauriers J, Nguyen BN, Huet PM (2002). Assessment of fatigue in patients with chronic hepatitis C using the Fatigue Impact Scale. Dig Dis Sci.

[5] Poynard T, Cacoub P, Ratziu V, Myers RP, Dezailles MH, Mercadier A, Ghillani P, Charlotte F, Piette JC, Moussalli J (2002). Fatigue in patients with chronic hepatitis C. J Viral Hepat.

[6] Kallman J, O’Neil MM, Larive B, Boparai N, Calabrese L, Younossi ZM (2007). Fatigue and health-related quality of life (HRQL) in chronic hepatitis C virus infection. Dig Dis Sci.

[7] Sarkar S, Jiang Z, Evon DM, Wahed AS, Hoofnagle JH (2012). Fatigue before, during and after antiviral therapy of chronic hepatitis C: results from the Virahep-C study. J Hepatol.

[8] McDonald J, Jayasuriya J, Bindley P, Gonsalvez C, Gluseska S (2002). Fatigue and psychological disorders in chronic hepatitis C. J Gastroenterol Hepatol.

[9] Weissenborn K, Krause J, Bokemeyer M, Hecker H, Schuler A, Ennen JC, Ahl B, Manns MP, Boker KW (2004). Hepatitis C virus infection affects the brain-evidence from psychometric studies and magnetic resonance spectroscopy. J Hepatol.

[10] Laskus T, Radkowski M, Adair DM, Wilkinson J, Scheck AC, Rakela J (2005). Emerging evidence of hepatitis C virus neuroinvasion. AIDS.

[11] Seaman K, Paterson BL, Vallis M, Hirsch G, Peltekian KM (2009). Future directions for investigation of fatigue in chronic hepatitis C viral infection. Chronic Illn.

[12] McHutchison JG, Lawitz EJ, Shiffman ML, Muir AJ, Galler GW, McCone J, Nyberg LM, Lee WM, Ghalib RH, Schiff ER, Galati JS, Bacon BR, Davis MN, Mukhopadhyay P, Koury K, Noviello S, Pedicone LD, Brass CA, Albrecht JK, Sulkowski MS (2009). Peginterferon alfa-2b or alfa-2a with ribavirin for treatment of hepatitis C infection. N Engl J Med.

[13] Casey LC, Lee WM (2012). Hepatitis C therapy update. Curr Opin Gastroenterol.

[14] Hezode C, Forestier N, Dusheiko G, Ferenci P, Pol S, Goeser T, Bronowicki JP, Bourliere M, Gharakhanian S, Bengtsson L, McNair L, George S, Kieffer T, Kwong A, Kauffman RS, Alam J, Pawlotsky JM, Zeuzem S (2009). Telaprevir and peginterferon with or without ribavirin for chronic HCV infection. N Engl J Med.

[15] Jacobson IM, Pawlotsky JM, Afdhal NH, Dusheiko GM, Forns X, Jensen DM, Poordad F, Schulz J (2012). A practical guide for the use of boceprevir and telaprevir for the treatment of hepatitis C. J Viral Hepat.

[16] Manns MP, McHutchison JG, Gordon SC, Rustgi VK, Shiffman M, Reindollar R, Goodman ZD, Koury K, Ling M-H, Albrecht JK (2001). Peginterferon alfa-2b plus ribavirin compared with interferon alfa-2b plus ribavirin for initial treatment of chronic hepatitis C: a randomised trial. Lancet.

[17] Fried MW, Shiffman ML, Reddy KR, Smith C, Marinos G, Gonçales FL, Häussinger D, Diago M, Carosi G, Dhumeaux D, Craxi A, Lin A, Hoffman J, Yu J (2002). Peginterferon alfa-2a plus ribavirin for chronic hepatitis C virus infection. N Engl J Med.

[18] Hassanein T, Cooksley G, Sulkowski M, Smith C, Marinos G, Lai MY, Pastore G, Trejo-Estrada R, Horta E Vale A, Wintfeld N, Green J (2004). The impact of peginterferon alfa-2a plus ribavirin combination therapy on health-related quality of life in chronic hepatitis C. J Hepatol.

[19] Asselah T, Marcellin P (2012). Direct acting antivirals for the treatment of chronic hepatitis C: one pill a day for tomorrow. Liver Int.

[20] Bacon BR, Gordon SC, Lawitz E, Marcellin P, Vierling JM, Zeuzem S, Poordad F, Goodman ZD, Sings HL, Boparai N, Burroughs M, Brass CA, Albrecht JK, Esteban R (2011). Boceprevir for previously treated chronic HCV genotype 1 infection. N Engl J Med.

[21] Poordad F, McCone J, Bacon BR, Bruno S, Manns MP, Sulkowski MS, Jacobson IM, Reddy KR, Goodman ZD, Boparai N, DiNubile MJ, Sniukiene V, Brass CA, Albrecht JK, Bronowicki JP (2011). Boceprevir for untreated chronic HCV genotype 1 infection. N Engl J Med.

[22] Fried MW, Buti M, Dore GJ, Flisiak R, Ferenci P, Jacobson I, Marcellin P, Manns M, Nikitin I, Poordad F, Sherman M, Zeuzem S, Scott J, Gilles L, Lenz O, Peeters M, Sekar V, De Smedt G, Beumont-Mauviel M (2013). Once-daily simeprevir (TMC435) with pegylated interferon and ribavirin in treatment-naive genotype 1 hepatitis C: the randomized PILLAR study. Hepatology.

[23] Zeuzem S, Berg T, Gane E, Ferenci P, Foster GR, Fried MW, Hézode C, Hirschfield GM, Jacobson I, Nikitin I, Pockros P, Poordad F, Scott J, Lenz O, Peeters M, Sekar V, De Smedt G, Sinha R, Beumont-Mauviel M (2014). Simeprevir (TMC435) in treatment-experienced HCV genotype 1 patients; the phase IIb, randomized. Controlled ASPIRE trial. Gastroenterology.

[24] Kleinman L, Mannix S, Yuan Y, Kummer S, L’Italien G, Revicki D (2012). Review of patient-reported outcome measures in chronic hepatitis C. Health Qual Life Outcomes.

[25] Rosa K, Fu M, Gilles L, Cerri K, Peeters M, Bubb J, Scott J (2014). Validation of the Fatigue Severity Scale in chronic hepatitis C. Health Qual Life Outcomes.

[26] Krupp LB, LaRocca NG, Muir-Nash J, Steinberg AD (1989). The fatigue severity scale. Application to patients with multiple sclerosis and systemic lupus erythematosus. Arch Neurol.

[27] Rabin R, de Charro F (2001). EQ-5D: a measure of health status from the EuroQol Group. Ann Med.

[28] Friedman JH, Alves G, Hagell P, Marinus J, Marsh L, Martinez-Martin P, Goetz CG, Poewe W, Rascol O, Sampaio C, Stebbins G, Schrag A (2010). Fatigue rating scales critique and recommendations by the Movement Disorders Society task force on rating scales for Parkinson’s disease. Mov Disord.

[29] Gencay-Can A, Can SS (2012). Validation of the Turkish version of the fatigue severity scale in patients with fibromyalgia. Rheumatol Int.

[30] Kleinman L, Zodet MW, Hakim Z, Aledort J, Barker C, Chan K, Krupp L, Revicki D (2000). Psychometric evaluation of the fatigue severity scale for use in chronic hepatitis C. Qual Life Res.

[31] Rasenack J, Zeuzem S, Feinman SV, Heathcote EJ, Manns M, Yoshida EM, Swain MG, Gane E, Diago M, Revicki DA, Lin A, Wintfeld N, Green J (2003). Peginterferon alpha-2a (40kD) [Pegasys] improves HR-QOL outcomes compared with unmodified interferon alpha-2a [Roferon-A]: in patients with chronic hepatitis C. Pharmacoeconomics.

[32] Blackburn S, Humphrey L, McCool R, Panter C, Young V, Peterson S, Mitchell L, Machouf N, Scott J: Interviews with patients with chronic hepatitis C virus infection document unmet needs, content validity, and comprehension of PROs for Clinical Trials. Poster presented at the 18th Annual Meeting of the International Society for Pharmacoeconomics and Outcomes Research, May 18-22, 2013, New Orleans, LA, USA

[33] Bernstein D, Kleinman L, Barker CM, Revicki DA, Green J (2002). Relationship of health-related quality of life to treatment adherence and sustained response in chronic hepatitis C patients. Hepatology.

[34] Versteegh MM, Rowen D, Brazier JE, Stolk EA (2010). Mapping onto EQ-5D for patients in poor health. Health Qual Life Outcomes.

[35] Szende A, Oppe M, Devlin N (2007). EQ-5D Value Sets: Inventory, Comparative Review and User Guide.

[36] Scott J, Gilles L, Fu M, Peeters M, Brohan E, Amatya R, Jessner W, Beumont-Mauviel M: Adding simeprevir to pegylated interferon-α/ribavirin for HCV shortens time with patient-reported symptoms and impairment in quality of life: results from the simeprevir Phase III QUEST-1, QUEST-2 and PROMISE studies. Poster presented at the 64th Annual Meeting of the American Association for the Study of Liver Diseases, Nov 1-5, 2013, Washington, DC, USA

[CR37] The pre-publication history for this paper can be accessed here:http://www.biomedcentral.com/1471-2334/14/465/prepub

