# Supplementary material for: Fatigue during treatment for hepatitis C virus: results of self-reported fatigue severity in two Phase IIb studies of simeprevir treatment in patients with hepatitis C virus genotype 1 infection
Source: BMC Infect Dis. 2014 Aug 26;14:465. doi: 10.1186/1471-2334-14-465 (PMC4162924; doi:10.1186/1471-2334-14-465)
Supplement: Supplementary file 1 — Additional file 1: Ethics Review Committees providing approval for PILLAR and ASPIRE.(PDF 129 KB) [file 12879_2013_3786_MOESM1_ESM.pdf]

Ethics approval for PILLAR received from the following 57 Ethics Review Committees between 30 March 2009 and 28 September 2009

**Ethics Review Committee**	**Investigators**
Comite De Etica De Protocolos De Investigacion<br>Gascón 450<br>Buenos Aires C1181ACH<br>Argentina	GALDAME, Omar Andres
Comite De Bioetica Del Hospital Francisco J Muniz<br>Uspallata 2272<br>Buenos Aires C1282AEN<br>Argentina	FAINBOIM, Hugo
Comite De Docencia Institucional<br>Billinghurst 1677 3 Piso<br>Buenos Aires C1425DTG<br>Argentina	TERG, Ruben
Comite Institucional de Evaluacion Universidad Austral<br>Avenida Juan Domingo Peron 1500<br>DERQUI - PILAR<br>Buenos Aires 1635<br>Argentina	SILVA, Marcelo Oscar
Comite De Bioetica Para Investigacion Clinica<br>Avenida Belgrano 1746<br>Buenos Aires 1093<br>Argentina	VILLAMIL, Federico
Sswahs Ethics Review Committee<br>Hospital Road<br>Concord NSW 2139<br>Australia	DORE, Greg<br>GEORGE, Jacob
Human Research Ethics Committee<br>St Vincent's Hospital<br>Victoria Pde<br>Fitzroy North 3065<br>Australia	DESMOND, Paul
Alfred Human Research & Ethics Committee<br>Alfred Hospital<br>Commercial Rd<br>Melbourne VIC 3004<br>Australia	ROBERTS, Stuart

Princess Alexandra Hospital HREC
Ipswich Road -
Woolloongabba
Brisbane N/A QLD 4102
Australia

MACDONALD, Graeme
LEE, Alice

Ethikkommission d. Med. Universität Wien und des Allgemeinen Krankenhauses der Stadt Wien
Borschkegasse 8B/E 06
Wien 1090
Austria

FERENCI, Peter
LAFERL, Hermann
GSCHWANTLER, Michael

Commision Biomedicale D Ethique Hospitalsfacultaire
Avenue Hippocrate Ucl 5514
Bruxelles 1200
Belgium

HORSMANS, Yves

Comité voor Medische Ethiek UZA
Wilrijkstraat 10
EDEGEM 2650
Belgium

MICHIELSEN, Peter

Commissie Medische Ethiek UZ Gasthuisberg Leuven
Herestraat 49.
Leuven 3000
Belgium

NEVENS, Frederik

Academisch Ziekenhuis Vub Ethische Commissie
Laarbeeklaan 101
Brussels 1090
Belgium

REYNAERT, Hendrik

Ethisch Comité Universitair Ziekenhuis Gent
De Pintelaan 185
Gent 9000
Belgium

VAN VLIERBERGHE, Hans

Commissie Voor Ethiek AZ St. Jan AV
Ruddershove 10
Brugge 8000
Belgium

ORLENT, Hans

Comite d'ethique Hospital Erasme
Route de Lennik 808
Anderlecht 1070
Belgium

MORENO, Christophe

H.-Hartziekenhuis Medisch-Ethische Commissie

DECAESTECKER, Jochen

Wilgenstraat 2
Roeselare 8800
Belgium

Comite de Etica em Pesquisa da PUCCamp<br>Rodovia Dom Pedro I km 136<br>Campinas SP<br>Brazil	LIMA, Maria Patelli
Comite De Etica Em Pesquisa-Faculdade De Ciencias Medicas-Unicamp<br>PO Box: 6111<br>Campinas SP 13083-970<br>Brazil	GONCALES JR, Fernando Lopes
Comite De Etica Em Pesquisa Da Maternidade Climerio De Oliveira<br>Rua do Limoeiro, 137 Nazaré<br>Salvador<br>Brazil	PARANA, Raymundo
Comite De Etica Em Pesquisa Do Hospital Sao Rafael<br>Av S Rafael 2152<br>Salvador<br>Brazil	LYRA, Luiz Guilherme
Comite De Etica Em Pesquisa Da FMUSP/HC<br>Rua Dr Ovídio Pires De Campos 225- Cerqueira César<br>Sao Paulo 05403-010<br>Brazil	PESSOA, Mario
McGill University Health Centre Research Ethics Board<br>1650, av. Cedar<br>Montréal QC H3G 1A4<br>Canada	GHALI, Maged Peter
Veritas IRB<br>1255 Transcanada Hwy Suite<br>310 Dorval<br>Montreal QC H9P 2V4<br>Canada	CÔTÉ, Pierre
Conjoint Health Research Ethics Board<br>3330 Hospital Dr Nw Room 93<br>Calgary AB T2N 4N1<br>Canada	LEE, Samuel
University Health Network Research Ethics Board	HEATHCOTE, Jenny

700 University<br>Toronto ON M5G 1Z5<br>Canada	HIRSCHFIELD, Gideon<br>SHERMAN, Morris
Den Videnskabsetiske Komite for Region Syddanmark<br>Regionshuset Damhaven 12<br>Vejle 7100<br>Denmark	GERSTOFT, Jan,MD<br>CHRISTENSEN, Peer Brehm<br>PEDERSEN, Court<br>LAURSEN, Alex Lund<br>MATHIESEN, Lars<br>MOELLER, Axel
CPP Ile De France III - Hopital Tarnier Cochin<br>89 rue D'Assas<br>Paris N/A 75006<br>France	BENHAMOU, Yves<br>BRONOWICKI, JeanPierre<br>GRANGE, JeanDidier<br>HEZODE, Christophe<br>LEBRAY, Pascal<br>MARCELLIN, Patrick<br>TRAN, Albert<br>TREPO, Christian<br>ZARSKI, Jean Pierre
Landesamt für Gesundheit und Soziales Berlin<br>Geschaeftsstelle der Ethik-Kommission des Landes Berlin<br>Fehrbellinerplatz<br>Berlin 10707<br>Germany	ARASTÉH, Keikawus<br>BERG, Thomas
Ethik-Kommission Der Medizinischen Fakultät Der Universität Würzburg<br>Institut für Pharmakologie und Toxikologie<br>Versbacher Strasse 9<br>Würzburg 97087<br>Germany	KLINKER, Hartwig,
Ethikkommission Bei Der Landesärztekammer Baden-Württemberg<br>Jahnstrasse 40<br>Stuttgart 70597<br>Germany	TREIN, Andreas,
Ethik-Kommission Der Medizinischen Fakultät Der Universität Zu Köln<br>Gleueler Str. 70<br>Geb. 5, Eingang A, Etage 2A,<br>Raum 015<br>Köln 50931<br>Germany	GOESER, Tobias

Ethik-Kommission Der Medizinische Hochschule Hannover<br>Carl-Neuberg-Strasse 1<br>Hannover 30625<br>Germany	MANNS, Michael Peter
Ethik-Kommission Der Ärztekammer Nordrhein<br>Tersteegenstr 9<br>Düsseldorf 40474<br>Germany	MAUSS, Stefan
Ethik-Kommission Der Albert Ludwigs Universität Freiburg<br>Engelbergerstr 21<br>Freiburg 79106<br>Germany	RASENACK, Jens
Ethik-Kommission des Fachbereichs Medizin der Johann-Wolfgang-Goethe Universität<br>Theodor-Stern-Kai 7<br>HAUS 1<br>Frankfurt 60590<br>Germany	ZEUZEM, Stefan
Ethik-Kommission der Aerztekammer Hamburg<br>Humboldtstr. 67a<br>22083 Hamburg<br>Germany	BUGGISCH, Peter<br>STELLBRINK, Hans Jurgen
Multi Region Ethics Committee<br>133 Molesworth Street<br>Wellington<br>New Zealand	STEDMAN, Catherine<br>DICKSON, Graeme<br>GANE, Ed<br>WEILERT, Frank
Reg. komite med. Og helsefaglig forskningsetikk<br>Sør-Øst-Norge (REK Sør-Øst)<br>**Post Address:** PO box 1130,<br>Blindern, 0318 Oslo, Norway<br>**Visit address**: Gullhaug torg<br>4a, 0484 Oslo, Norway	BRUUN, Trond<br>VON DER LIPPE, Bent<br>KONOPSKI, Zbigniew<br>BLOCK HELLUM, Kjell<br>FLORHOLMEN, Jon<br>LANGELAND, Nina
Komisja Bioetyczna Uniwersytetu Medycznego w Bialymstoku<br>Ul Kilinskiego 1<br>Bialystok 15-089<br>Poland	FLISIAK, Robert<br>HASSMANNPOZNANSKA, Elzbieta<br>HORBAN, Andrzej<br>HALOTA, Waldemar<br>KRYCZKA, Wieslaw<br>JABLKOWSKI, Maciej<br>JANCZEWSKA-KAZEK, Ewa
Independent Ethics Committee Of Smolensk Regional Clinical Hospital	RAFALSKIY, Vladimir

27 Gagarina Avenue<br>Smolensk 214019<br>Russia	
Independent Ethics Committee Of Moscow<br>Regional Clinical Institute Vladimirsky<br>61/2 Schepkina Street<br>Moscow 129111<br>Russia	BOGOMOLOV, Pavel
Interuniversity Ethics Committee And Association Of Medical And Pharmaceutical Institute Of Higher Education<br>8 Build 2 Trubetskaya Str<br>Moscow 119992<br>Russia	IVASHKIN, Vladimir
Independent Interdisciplinary. Committee On Ethical Exploration of Clinical Studies<br>51 Leningradsky Prospekt<br>Moscow 125468<br>Russia	KOROCHKINA, Olga<br>MOROZOV, Vyacheslav<br>NIKITIN, Igor G<br>YAKOVLEV, Alexey,<br>VORONIN, Evgeniy<br>ZAKHAROVA, Natalia
Comite Etico De Investigacion Clinica Del Hospital<br>Universitari Vall D'Hebron - 2A Planta<br>Paseo Vall D'Hebron, 119-129<br>Institut De Recerca Hospital Universitari Vall D'Hebron<br>Barcelona 08035<br>Spain	MORENO OTERO, Ricardo<br>CALLEJA, Jose Luis<br>DIAGO, Moises<br>ROMERO, Manuel<br>BUTI, Maria
Hospital La Princesa - Comite Etico De Investigacion Clinica<br>Diego De Leon 62<br>Madrid 28006<br>Spain	MORENO OTERO, Ricardo
Ec Of Hospital De Valme<br>Carretera De Cadiz S/n Km 548 9<br>Sevilla N/a 41014<br>Spain	BUTI, Maria<br>ROMERO, Manuel
CEIC AREA 6 - Hospital Universitario Puerta De Hierro De Majadahonda<br>C/ Joaquin Rodrigo, 2<br>Majadahonda (Madrid) 28222<br>Spain	CALLEJA, Jose Luis

CEIC Hospital La Ribera de Alzira<br>Corbera<br>Alzira VALENCIA 46600<br>Spain	DIAGO, Moises
Ochsner Institutional Review Board<br>1514 Jefferson Hwy<br>Biomedical Research Bldg.<br>New Orleans LA 70121<br>USA	GIRGRAH, Nigel
IRB Of Cedars-Sinai Medical Center<br>8383 Wilshire Blvd<br>SUITE 742<br>Beverly Hills CA 90211<br>USA	POORDAD, Fred
Committee On Human Rights In Research<br>Weill Medical College Of Cornell University<br>425 E 61St St Rm 301<br>New York NY 10021<br>USA	JACOBSON, Ira M
Office of Human Research Ethics<br>School of Medicine Bldg. #52, CB 7097<br>The University of North Carolian at Chapel Hill<br>Chapel Hill, NC 27599-7097<br>USA	FRIED, Michael
University Of Chicago Office Of Clinical Research<br>Section Regulatory Compliance<br>5751 S Woodlawn Ave<br>McGiffert Hall, 2nd floor<br>Chicago IL 60637<br>USA	JENSEN, Donald M
Sterling Institutional Review Board<br>6300 Powers Ferry Rd, Suite 600-351<br>Atlanta GA 30339<br>USA	DE JESUS, Edwin<br>ETZKORN, Kyle<br>JONAS, Mark<br>PAMBIANCO, Daniel<br>SMITH, Coleman<br>TAUNK, Jawahar<br>WRUBLE, Lawrence<br>YOUNES, Ziad

Ethics Approval for ASPIRE received from the following 54 Ethics Review Committees between 17 September 2009 and 24 December 2009

**Ethics Review Committee**	**Investigators**
Human Research Ethics Committee<br>St Vincent's Hospital<br>Victoria Pde<br>Fitzroy North 3065<br>Australia	DESMOND, Paul
Alfred Human Research & Ethics Committee<br>Alfred Hospital<br>Commercial Rd<br>Melbourne VIC 3004<br>Australia	ROBERTS, Stuart<br>GEORGE, Jacob
Princess Alexandra Hospital HREC<br>Ipswich Road -<br>Woolloongabba<br>Brisbane N/A QLD 4102<br>Australia	MACDONALD, Graeme
St. Vincent's Ethics Committee<br>390 Victoria Straat<br>St Vincent's Hospital Sydney Ltd<br>Darlinghurst NSW 2010<br>Australia	DORE, Greg<br>HUGHES, Brian<br>LEE, Alice
Melbourne Health Research Directorate<br>Royal Melbourne Hospital<br>Grattan Street<br>Parkville - VIC VIC 3050<br>Australia	NICOLL, Amanda
Ethikkommission d. Med. Universität Wien und des Allgemeinen Krankenhauses der Stadt Wien<br>Borschkegasse 8B/E 06<br>Wien 1090<br>Austria	FERENCI, Peter<br>LAFERL, Hermann<br>GSCHWANTLER, Michael
Comité d’Ethique UCL Saint-Luc<br>10 Avenue Hippocrate<br>UCL Saint Luc<br>Brussels 1200<br>Belgium	HORSMANS, Yves
Comité voor Medische Ethiek UZA<br>Wilrijkstraat 10	MICHIELSEN, Peter

Edegem 2650<br>Belgium	
Commissie Medische Ethiek UZ Gasthuisberg<br>Herestraat 49<br>Leuven 3000<br>Belgium	NEVENS, Frederik
Academisch Ziekenhuis Vub Ethische Commissie<br>Laarbeeklaan 101<br>Brussels 1090<br>Belgium	REYNAERT, Hendrik
Ethisch Comité Universitair Ziekenhuis Gent<br>De Pintelaan 185<br>Gent 9000<br>Belgium	VAN VLIERBERGHE, Hans
Commissie Voor Ethiek AZ<br>St. Jan AV<br>Ruddershove 10<br>BRUGGE 8000<br>Belgium	ORLENT, Hans
Comite d'ethique ULB<br>Hospital Erasme<br>Route de Lennik 808<br>Anderlecht 1070<br>Belgium	MORENO, Christophe
H.-Hartziekenhuis Medisch-Ethische Commissie<br>Wilgenstraat 2<br>Roeselare 8800<br>Belgium	DECAESTECKER, Jochen
Ottawa Hospital Research Ethics Board<br>751 Parkdale Ave Suite 106<br>Civic Campus<br>Ottawa ON K1Y 1J7<br>Canada	COOPER, Curtis
McGill University Health Centre Research Ethics Board<br>1650, av. Cedar<br>Montréal QC H3G 1A4<br>Canada	GHALI, Maged Peter
Veritas IRB<br>1255 Transcanada Hwy Suite	CÔTÉ, Pierre<br>VEZINA, Sylvie

310 Dorval<br>Montreal QC H9P 2V4<br>Canada	
Conjoint Health Research Ethics Board<br>3330 Hospital Dr Nw Room 93<br>Calgary AB T2N 4N1<br>Canada	MYERS, Rob
University Health Network Research Ethics Board<br>700 University<br>Toronto ON M5G 1Z5<br>Canada	HEATHCOTE, Jenny<br>FUNG, Scott
CPP Est I<br>Faculte De Medecine<br>BP 87900<br>Dijon Cedex 21079<br>France	BENHAMOU, Yves<br>BRONOWICKI, Jean Pierre<br>GRANGE, Jean Didier<br>HEZODE, Christophe<br>LEBRAY, Pascal<br>SERFATY, Lawrence<br>TRAN, Albert<br>ZARSKI, Jean Pierre<br>ZOULIM, Fabien
Landesamt für Gesundheit und Soziales Berlin<br>Geschaeftsstelle der Ethik-Kommission des Landes Berlin<br>Fehrbellinerplatz<br>Berlin 10707<br>Germany	ARASTÉH, Keikawus<br>BERG, Thomas
Ethik-Kommission Der Medizinischen Fakultät Der Universität Würzburg<br>Institut für Pharmakologie und Toxikologie<br>Versbacher Strasse 9<br>Würzburg 97087<br>Germany	KLINKER, Hartwig
Ethikkommission Bei Der Landesärztekammer Baden-Württemberg<br>Jahnstrasse 40<br>Stuttgart 70597<br>Germany	TREIN, Andreas
Ethik-Kommission Der Medizinischen Fakultät Der Universität Zu Köln<br>Gleueler Str. 70<br>Geb. 5, Eingang A, Etage 2A,<br>Raum 015	GOESER, Tobias

Köln 50931
Germany

Ethik-Kommission Der Medizinische Hochschule Hannover<br>Carl-Neuberg-Strasse 1<br>Hannover 30625<br>Germany	MANNS, Michael Peter
Ethik-Kommission Der Ärztekammer Nordrhein<br>Tersteegenstr 9<br>Düsseldorf 40474<br>Germany	MAUSS, Stefan
Ethik-Kommission Der Albert Ludwigs Universität Freiburg<br>Engelbergerstr 21<br>Freiburg 79106<br>Germany	RASENACK, Jens
Ethik-Kommission des Fachbereichs Medizin der<br>Johann-Wolfgang-Goethe Universität<br>Theodor-Stern-Kai 7<br>HAUS 1<br>Frankfurt 60590<br>Germany	ZEUZEM, Stefan
Ethik-Kommission der Aerztekammer Hamburg<br>Humboldtstr. 67a<br>22083 Hamburg<br>Germany	BUGGISCH, Peter
Charing Cross-Hospital Ethics Committee<br>London - Bloomsbury<br>Charing Cross Hospital,<br>Room 4W/12, 4th Floor<br>Fulham Palace Road<br>London W6 8RF<br>Great Britain	AGARWAL, Kaushik<br>FOSTER, Graham R<br>BROWN, Ashley<br>CRAMP, Matthew
Helsinki Committee<br>Rambam Medical Center<br>Ha'aliyah Street Bat Gallm<br>Haifa 31096<br>Israel	BARUCH, Yaacov
Helsinki Committee<br>Sourasky Medical Center<br>6 Weitzman Street<br>Tel Aviv 64239	LURIE, Yoav

Israel	
Helsinki Committee<br>Hadassah Medical Center<br>Ein Carem<br>Jerusalem 91120<br>Israel	SHOUVAL, Daniel
Helsinki Committee<br>Lady Davis Carmel Medical Center<br>7 MICHAL STREET<br>HAIFA 34362<br>Israel	ZUCKERMAN, Elimelech
Helsinki Committee<br>Rebecca Ziv Medical Center<br>Darom Neighborhood<br>Zefat 13000<br>Israel	NIMER, Assy
Helsinki Committee<br>Rabin Medical Center<br>Kaplan Street<br>Petah Tiqva 49100<br>Israel	BEN-ARI, Ziv
Helsinki Committee<br>Chaim Sheba Medical Center<br>Tel Hashomer<br>Ramat Gan 52621<br>Israel	MAOR, Yaaov
Helsinki Committee<br>Holy Family Medical Center<br>Nazareth<br>Israel	SAFADI, Rifaat
Multi Region Ethics Committee<br>133 Molesworth Street<br>Wellington<br>New Zealand	STEDMAN, Catherine<br>DICKSON, Graeme<br>GANE, Ed
Reg. komite med. Og helsefaglig forskningsetikk<br>Sør-Øst-Norge (REK Sør-Øst)<br>**Post Address:** PO box 1130,<br>Blindern, 0318 Oslo, Norway<br>**Visit address**: Gullhaug torg<br>4a, 0484 Oslo, Norway	VON DER LIPPE, Bent<br>KONOPSKI, Zbigniew<br>BLOCK HELLUM, Kjell<br>FLORHOLMEN, Jon<br>LANGELAND, Nina

Komisja Bioetyczna Uniwersytetu Medycznego w Bialymstoku<br>Ul Kilinskiego 1<br>Bialystok 15-089<br>Poland	FLISIAK, Robert<br>HASSMANNPOZNANSKA, Elzbieta<br>HORBAN, Andrzej<br>HALOTA,Waldemar<br>KRYCZKA,Wieslaw<br>JABLKOWSKI, Maciej<br>JANCZEWSKA-KAZEK, Ewa
Comissão de Ética para a Investigação Científica<br>Parque de Saúde de Lisboa<br>Avenida Do Brasil 53<br>Pav. 17 - A<br>Lisboa 1749-004<br>Portugal	PEIXE, Paula<br>CALINAS, Antonio Filipe<br>CARVALHO, Armando<br>MACEDO, Guilherme<br>SEREJO, Fatima
Independent Ethics Committee Of Smolensk Regional Clinical Hospital<br>27 Gagarina Avenue<br>Smolensk 214019<br>Russia	RAFALSKIY, Vladimir
Independent Ethics Committee Of Moscow<br>Regional Clinical Institute Vladimirsky<br>61/2 Schepkina Street<br>Moscow 129111<br>Russia	BOGOMOLOV, Pavel
Interuniversity Ethics Committee And Association Of Medical And Pharmaceutical Institute Of Higher Education<br>8 Build 2 Trubetskaya Str<br>Moscow 119992<br>Russia	IVASHKIN, Vladimir
Independent Interdisciplinary. Committee On Ethical Exploration of Clinical Studies<br>51 Leningradsky Prospekt<br>Moscow 125468<br>Russia	KOROCHKINA, Olga<br>MOROZOV, Vyacheslav<br>NIKITIN, Igor G<br>YAKOVLEV, Alexey<br>VORONIN, Evgeniy<br>ZAKHAROVA, Natalia
The Scripps Clinic IRB<br>11025 N. Torrey Pines Rd;<br>La Jolla, CA 92037<br>USA	POCKROS, Paul
Ochsner Institutional Review Board	GIRGRAH, Nigel

1514 Jefferson Hwy<br>Biomedical Research Bldg.<br>New Orleans LA 70121<br>USA	
IRB Of Cedars-Sinai Medical Center<br>8383 Wilshire Blvd<br>Suite 742<br>Beverly Hills CA 90211<br>USA	POORDAD, Fred
Committee On Human Rights<br>New York Presbyterian Hospital<br>Weill Medical College Of Cornell University<br>407 East 61ST Street, RR-110<br>New York NY 10065<br>USA	JACOBSON, Ira M
Committee On The Protection Of The Rights Of Human Subjects<br>School Of Medicine<br>The University Of North Carolina At Chapel Hill<br>Campus Box # 7097, Medical School Building # 52<br>Chapel Hill NC 27599-7097<br>USA	FRIED, Michael
University Of Chicago Office Of Clinical Research<br>Section Regulatory Compliance<br>5751 S Woodlawn Ave<br>McGiffert Hall, 2nd floor<br>Chicago IL 60637<br>USA	JENSEN, Donald M
Sterling Institutional Review Board<br>6300 Powers Ferry Rd, Suite 600-351<br>Atlanta GA 30339<br>USA	CHASEN, Richard<br>DE JESUS, Edwin<br>ETZKORN, Kyle<br>JONAS, Mark<br>LAWITZ, Eric<br>SCHWARTZ, Howard<br>STEIN, Ira<br>TAUNK, Jawahar<br>UNDERWOOD, James<br>WRUBLE, Lawrence<br>YOUNES, Ziad
North Mississippi Health Services IRB<br>830 South Gloster Street East<br>Tower 3Rd Floor<br>Tupelo MS 38801, USA	PHILIPS, John
