# Supplementary material for: Fatigue during treatment for hepatitis C virus: results of self-reported fatigue severity in two Phase IIb studies of simeprevir treatment in patients with hepatitis C virus genotype 1 infection
Source: BMC Infect Dis. 2014 Aug 26;14:465. doi: 10.1186/1471-2334-14-465 (PMC4162924; doi:10.1186/1471-2334-14-465)
Supplement: Supplementary file 2 — Authors’ original file for figure 1 [file 12879_2013_3786_MOESM2_ESM.pdf]

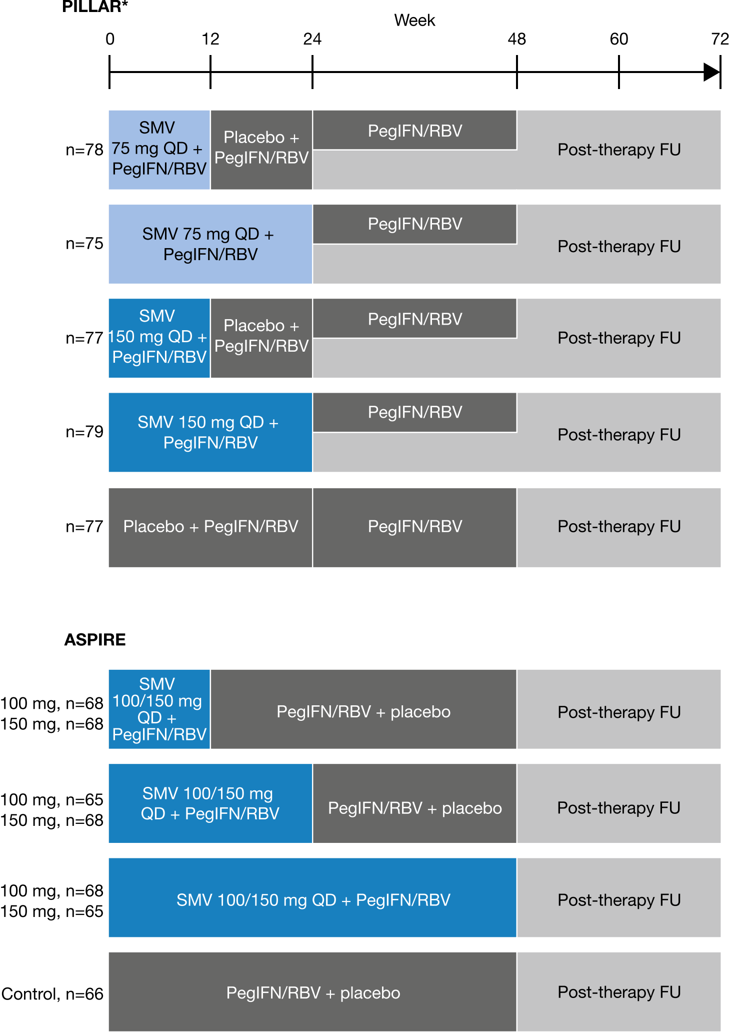

*RGT criteria in SMV arms; end treatment at Week 24 if HCV RNA <25 IU/mL detectable/undetectable at Week 4 and <25 IU/mL undetectable at Weeks 12, 18 and 20 (all other patients continued PegIFN/RBV up to Week 48).

SMV, simeprevir; QD, once-daily; PegIFN/RBV, peginterferon-α and ribavirin; FU, follow-up; HCV, hepatitis C virus.
