# Supplementary material for: Fatigue during treatment for hepatitis C virus: results of self-reported fatigue severity in two Phase IIb studies of simeprevir treatment in patients with hepatitis C virus genotype 1 infection
Source: BMC Infect Dis. 2014 Aug 26;14:465. doi: 10.1186/1471-2334-14-465 (PMC4162924; doi:10.1186/1471-2334-14-465)
Supplement: Supplementary file 3 — Authors’ original file for figure 2 [file 12879_2013_3786_MOESM3_ESM.pdf]

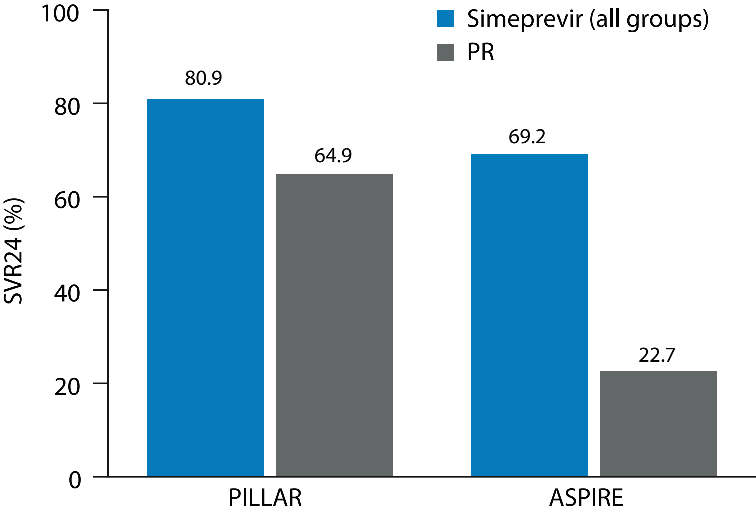

SVR24, sustained virological response defined as hepatitis C virus RNA<25 IU/mL and undetectable at 24 weeks after planned end of treatment; PR, placebo/peginterferon-α and ribavirin
