# Supplementary figures and images for: Fatigue during treatment for hepatitis C virus: results of self-reported fatigue severity in two Phase IIb studies of simeprevir treatment in patients with hepatitis C virus genotype 1 infection
Source: BMC Infect Dis. 2014 Aug 26;14:465. doi: 10.1186/1471-2334-14-465 (PMC4162924; doi:10.1186/1471-2334-14-465)

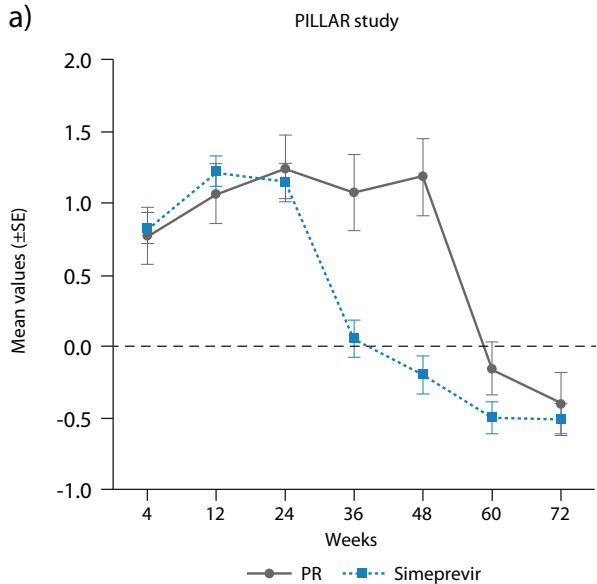

Number of patients

|            |     |     |     |     |     |     |     |
|------------|-----|-----|-----|-----|-----|-----|-----|
| PR         | 185 | 187 | 174 | 172 | 166 | 164 | 164 |
| Simeprevir | 49  | 46  | 48  | 45  | 43  | 43  | 44  |

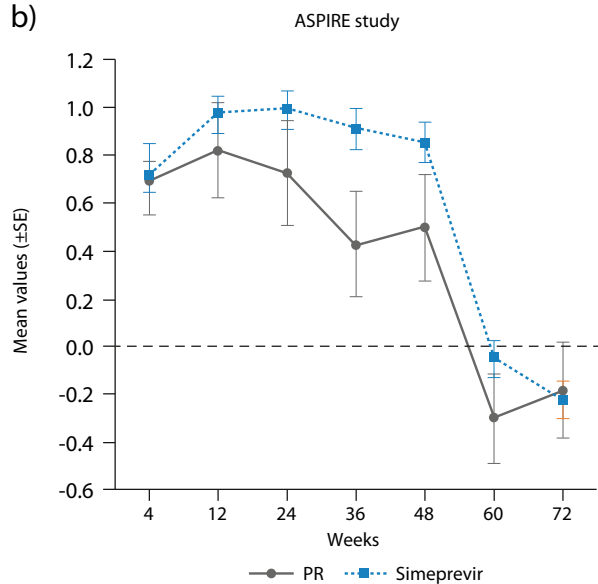

Number of patients

|            |     |     |     |     |     |     |     |
|------------|-----|-----|-----|-----|-----|-----|-----|
| PR         | 367 | 356 | 343 | 346 | 344 | 342 | 338 |
| Simeprevir | 61  | 54  | 52  | 53  | 52  | 54  | 56  |

Supplement: Supplementary file 4 — Authors’ original file for figure 3 [file 12879_2013_3786_MOESM4_ESM.pdf]

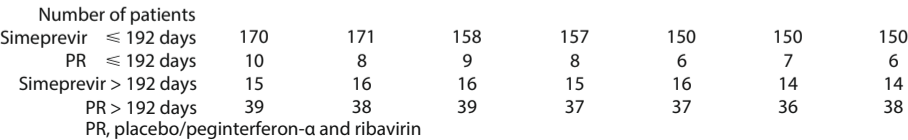

Supplement: Supplementary file 5 — Authors’ original file for figure 4 [file 12879_2013_3786_MOESM5_ESM.pdf]
