# Supplementary material for: Fatigue during treatment for hepatitis C virus: results of self-reported fatigue severity in two Phase IIb studies of simeprevir treatment in patients with hepatitis C virus genotype 1 infection
Source: BMC Infect Dis. 2014 Aug 26;14:465. doi: 10.1186/1471-2334-14-465 (PMC4162924; doi:10.1186/1471-2334-14-465)
Supplement: Supplementary file 6 — Authors’ original file for figure 5 [file 12879_2013_3786_MOESM6_ESM.pdf]

a)

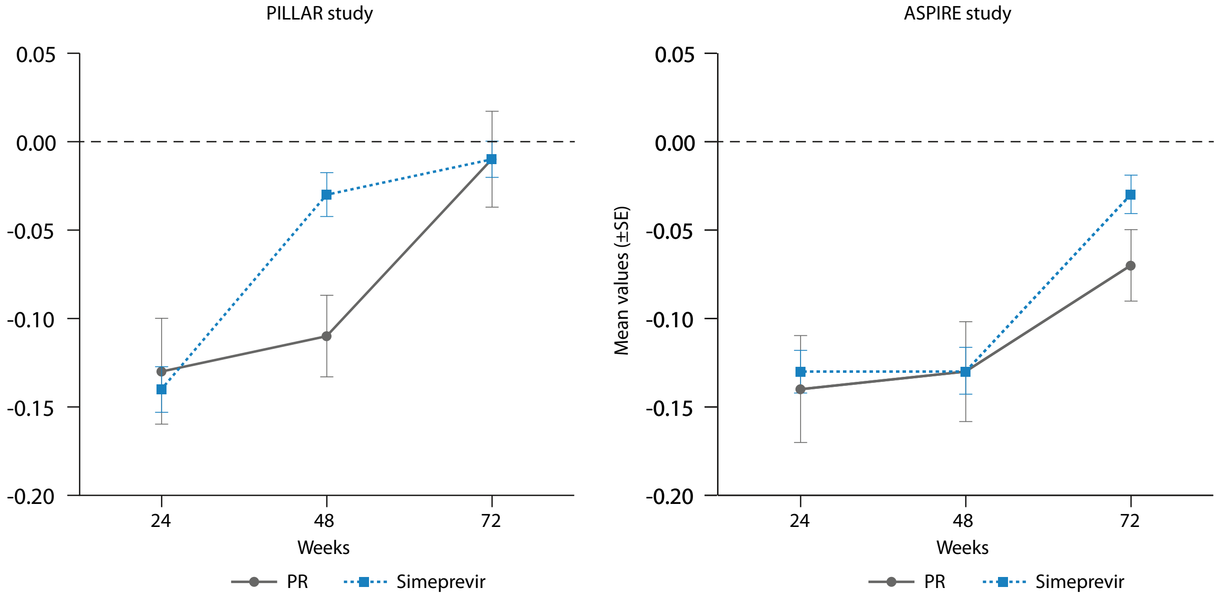

Number of patients (PILLAR study)

	24	48	72
PR	72	67	68
Simeprevir	279	268	275

Number of patients (ASPIRE study)

	24	48	72
PR	50	53	55
Simeprevir	344	342	330

b)

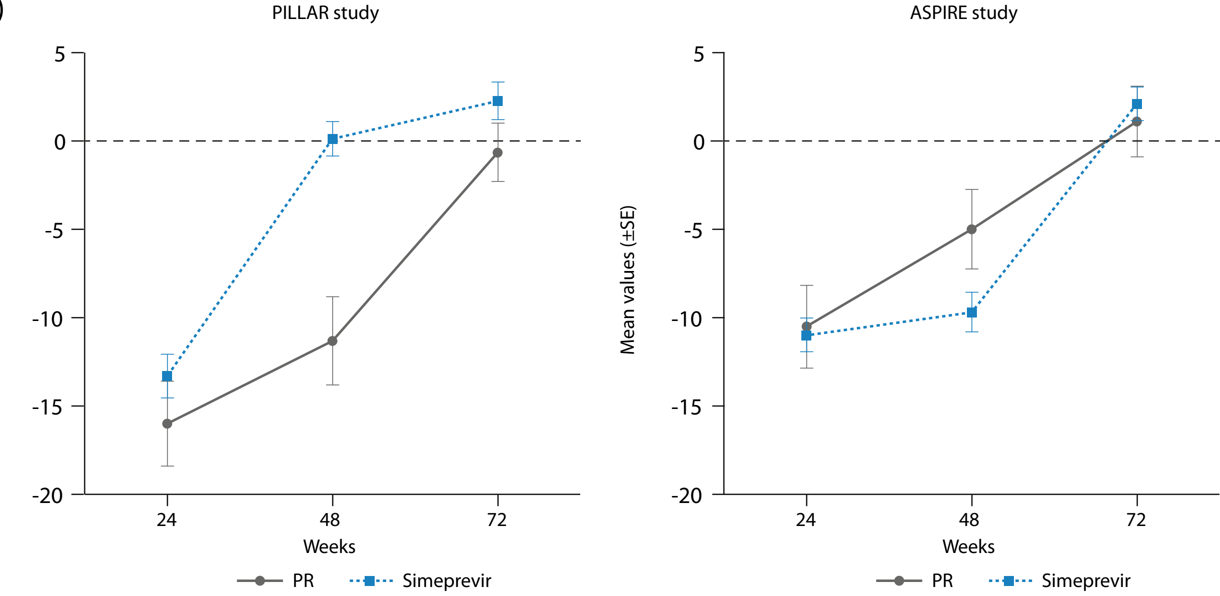

Number of patients (PILLAR study)

	24	48	72
PR	72	66	68
Simeprevir	275	269	274

Number of patients (ASPIRE study)

	24	48	72
PR	50	53	56
Simeprevir	342	341	331

EQ-5D, European Quality of Life 5-Dimensions; VAS, visual analog scale; PR, placebo/peginterferon-α and ribavirin; SE, standard error
