# Supplementary material for: Fatigue during treatment for hepatitis C virus: results of self-reported fatigue severity in two Phase IIb studies of simeprevir treatment in patients with hepatitis C virus genotype 1 infection
Source: BMC Infect Dis. 2014 Aug 26;14:465. doi: 10.1186/1471-2334-14-465 (PMC4162924; doi:10.1186/1471-2334-14-465)
Supplement: Supplementary file 7 — Authors’ original file for figure 6 [file 12879_2013_3786_MOESM7_ESM.pdf]

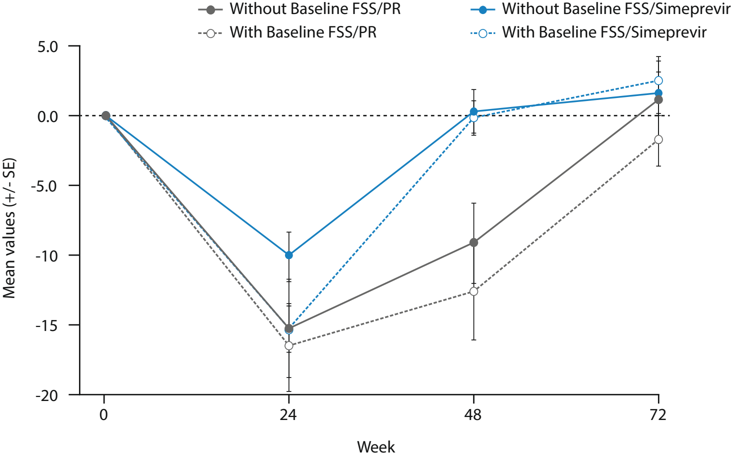

Number of patients				
Without Baseline FSS/PR	27	26	23	24
Without Baseline FSS/Simeprevir	112	102	100	102
With Baseline FSS/PR	49	46	43	44
With Baseline FSS/Simeprevir	193	173	169	172

PR, placebo/peginterferon-α and ribavirin
